# Identification of Cuticular and Web Lipids of the Spider *Argiope bruennichi*

**DOI:** 10.1007/s10886-021-01338-y

**Published:** 2022-01-10

**Authors:** Moritz Gerbaulet, Anton Möllerke, Katharina Weiss, Satya Chinta, Jutta M. Schneider, Stefan Schulz

**Affiliations:** 1 Institute of Organic Chemistry, TU Braunschweig, Hagenring 30, 38106 Braunschweig, Germany; 2 Institute of Zoology, Universität Hamburg, Martin-Luther-King Platz 3, 20146 Hamburg, Germany; 3 U.S. Department of Agriculture, Agricultural Research Service, 1600-1700 SW 23rd Drive, Gainesville, FL 32608 USA

**Keywords:** GC/MS, Wax esters, Branched fatty acids, Pheromones, Kin-recognition

## Abstract

Emerging evidence shows that the cuticular and silk lipids of spiders are structurally more diverse than those of insects, although only a relatively low number of species have been investigated so far. As in insects, such lipids might play a role as signals in various contexts. The wasp spider *Argiope bruennichi* has probably the best investigated chemical communication system within spiders, including the known structure of the female sex pheromone. Recently we showed that kin-recognition in *A. bruennichi* could be mediated through the cuticular compounds consisting of hydrocarbons and, to a much larger proportion, of wax esters. By use of mass spectrometry and various derivatization methods, these were identified as esters of 2,4-dimethylalkanoic acids and 1-alkanols of varying chain lengths, such as tetradecyl 2,4-dimethylheptadecanoate. A representative enantioselective synthesis of this compound was performed which proved the identifications and allowed us to postulate that the natural enantiomer likely has the (2*R*,4*R*)-configuration. Chemical profiles of the silk and cuticular lipids of females were similar, while male cuticular profiles differed from those of females. Major components of the male cuticular lipids were tridecyl 2,4-dimethyl-C_17-19_ alkanoates, whereas those of females were slightly longer, comprising tridecyl 2,4-dimethyl-C_19-21_ alkanoates. In addition, minor female-specific 4-methylalkyl esters were detected.

**Supplementary Information:**

The online version contains supplementary material available at 10.1007/s10886-021-01338-y.

## Introduction

Chemical communication is the predominant form of communication in arthropods because their typically small size limits the efficacy of, for example, visual or acoustic communication (Greenfield 2002). Often, cuticular lipids, besides their role as a water barrier (Gibbs and Rajpurohit 2010), have important communicative functions, as is well documented for insects (Howard and Blomquist 2005, Blomquist and Bagnères 2010). In arthropods other than insects, however, cuticular chemistry and its role in communication is largely unknown.

For example, in spiders, the chemistry of cuticular lipids and especially silk has been investigated in only a few species. Generally, the composition of cuticular lipids seems to be more divers in spiders than in insects. In a couple of previous studies it was shown that after alkanes, the dominating compound class within insects (Blomquist and Bagnères 2010), major components can be long-chain, methyl-branched methyl ethers. These have been reported from linyphiids (Schulz and Toft 1993), *Nephila clavipes* (Schulz 2001), and *Tetragnatha* sp. (Adams et al. 2021). Fatty acids and aliphatic saturated alcohols occur in various other species (Trabalon et al. 1996, 1997; Prouvost et al. 1999; Trabalon and Assi-Bessekon 2008; Trabalon 2011). These compounds can be combined to form esters. Such esters can be simple methyl esters of fatty acids (Prouvost et al. 1999; Trabalon 2011), but some species have evolved more elaborate compounds. The social spider *Anelosimus eximus* produces a complex array of long-chain methyl branched *n*-propyl esters, e. g. propyl 4,20-dimethylhentriacontanoate (Bagnères et al. 1997). Another ester type is present on the cuticle of the spider *Argyrodes elevatus* (Theridiidae), consisting of short, partly branched alcohols conjugated to longer acids, occurring in completely different sex-specific mixtures of a few compounds. These include undecyl 2-methyltridecanoate in males and 2,8-dimethylundecyl 2,8-dimethylundecanoate as well as heptadecyl 4-methylheptanoate in females. Other spiders exclusively use insect-type hydrocarbons (Trabalon and Assi-Bessekon 2008; Grinsted et al. 2011). As in insects, these cuticular constituents were suggested to transmit intraspecific information in various ways (Witte et al. 2009; Trabalon and Bagnères 2010; Xiao et al. 2010; Grinsted et al. 2011; Beeren et al. 2012; Schulz 2013; Ruhland et al. 2019; Adams et al. 2021), although compared to volatile pheromones in spiders (Schulz 2004, 2013; Gaskett 2007; Fischer 2019) this area is less well studied.

Among spiders, the chemical communication system of the European wasp spider *Argiope bruennichi* is probably one of the best-studied. As entelegyne spiders, genital structures are paired in both sexes, but only one can be used during a copulation (Foelix 2011). After sperm transfer, males effectively plug the genital opening of the female with parts of their genitalia that are rendered dysfunctional thereafter (Nessler et al. 2007; Uhl et al. 2010). Most males fall victim to sexual cannibalism after their first copulation, leaving one genital opening of the female available for another male. Males that escape and copulate twice can monopolize paternity with a female because both openings are securely plugged. Consequently, *A. bruennichi* females and males are restricted to a maximum of two copulations. Females are known to produce a volatile pheromone, trimethyl methylcitrate, that attracts males in the field and elicits courtship behavior (Chinta et al. 2010). Behavioral experiments indicate, however, that males also use the pheromone to assess a female`s condition, age, and mating status. For example, field observations (Welke et al. 2012) and laboratory experiments (Cory and Schneider 2016) demonstrated that males are more likely to monopolize females that are relatively heavy and old suggesting that females modulate pheromone release according to their state (Cory and Schneider 2016). Furthermore, mating experiments with siblings and non-siblings indicate that males are also capable of kin recognition. While male *A. bruennichi* readily copulate with sisters in laboratory mating trials, they terminate copulation earlier as compared to unrelated females (Welke and Schneider 2010). Consequently, they survive copulation more often (Welke and Schneider 2010), allowing them to leave and search for another female. Recently some of us showed that kin-recognition might be mediated by cuticular compounds occurring in family-specific bouquets and suggested an important role of unusual long-chain esters in kin recognition (Weiss and Schneider 2021). We present here the identification and synthesis of the dominant group of compounds of these bouquets, long chain esters of 2,4-dimethyl-branched acids with mostly unbranched fatty alcohols, and clarify their distribution.

## Methods and Materials

### Spider Extracts

Subadult male and female *Argiope bruennichi* (Scopoli, 1772) (Araneae, Araneidae) were collected from natural meadows in Northern Germany (Buxtehude, Harmstorf, Pevestorf, Lower Saxony; Wedel, Schleswig–Holstein), between 24 June and 5 July 2019. *Argiope bruennichi* is common throughout Europe and its collection requires no permits. Spiders were transferred to the laboratory at the University of Hamburg, Germany, where they were individually housed in upturned plastic cups (250 or 500 mL depending on the spider’s size) with a hole in the bottom stuffed with cotton wool. Spiders were kept under natural light conditions at a constant temperature of 25 °C and relative humidity of 45%. Spiders were checked for adult molts daily. In both sexes, subadult and adult spiders can be unambiguously distinguished by their genitalia (Uhl et al. 2007). A spider’s age is defined here as days since adult molt. Twice a week, subadult spiders were provided with approximately 15 *Drosophila* spp. and adult females with three *Calliphora* sp. houseflies. Adult males were fed with approximately 10 *Drosophila* spp. once a week. All spiders were provided with water from a sprayer at least six days a week. Twenty virgin male (mean age ± SD: 13.5 ± 1.2 days) and 30 virgin female *A. bruennichi* (mean age ± SD: 11.8 ± 0.9 days) were used for chemical analysis.

### Analysis by coupled gas chromatography-mass spectrometry (GC/MS)

To obtain silk samples, the females were placed into clean Perspex frames (35 × 35 × 6 cm) and allowed to build a web. After 24 h, spiders were removed from their webs and the silk was collected by slowly winding it around a glass Pasteur-pipet washed with ethanol. The tip of the pipet holding the silk was then snapped off into a small glass vial. Males and females were placed in clean glass vials and cold anesthetized. All samples were stored at − 25 °C until analysis. Cuticular extracts were prepared by placing individual females in 3 mL of dichloromethane (DCM; GC/MS grade, Merck, Darmstadt, Germany) and the smaller males in 1 mL DCM for one hr. Silk samples were extracted in 1 mL DCM. Extracts were concentrated by evaporation at room temperature to approximately 90 µl (females) and 50 µl (males and silk), respectively. An aliquot of 1 µl of each sample was analyzed by GC/MS on a Shimadzu GCMS-QP2010S system (Shimadzu Corporation, Kyoto, Japan). After individual analyses, the female silk and body extracts and male samples were pooled separately for further chemical identification. For the analysis of samples of individuals, the gas chromatograph was equipped with an SH-Rtx-5MS fused silica capillary column (30 × 0.25 mm ID, 0.25 µm film thickness; Shimadzu Corporation, Kyoto, Japan). The oven temperature was programmed from 80 to 260 °C at 30°/min and from 260 to 300 °C at 1°/min, with a 1-min initial isothermal and a 10-min final isothermal hold. A split-splitless injector was used at 250 °C in splitless mode. The carrier gas was helium at a constant flow rate of 1 mL/min. The ionization voltage of the electron ionization mass spectrometer was 70 eV. Source temperature was 200 °C and the interface temperature was 280 °C. Data acquisition and storage were performed with the software GCMSsolution (Version 4.45; Shimadzu Corporation, Kyoto, Japan). Peak areas were obtained by manual integration using the GCMSsolution software. Linear retention indices of all substances were calculated according to van den Dool and Kratz (1963). *n*-Alkanes were identified by comparing their mass spectra with those of authentic reference compounds. Alkenes were identified by their typical mass spectra. Methyl-branched hydrocarbons were identified by diagnostic ions resulting from their typical α-cleavage at the position of the methyl branch and by a fragment at M-15 if the molecular ion was not detected. Moreover, their linear retention indices were compared to published values (Carlson et al. 1998; El-Sayed 2019) and also to calculated theoretical values as described by Schulz (2001). Other compounds were tentatively identified by comparing their mass spectra and linear retention indices with those of a database (NIST 08 mass spectral library 2008). Mean relative peak areas were calculated by standardizing total mean peak areas to 100%. GC/MS analyses of pooled samples were performed on a GC 7890A coupled to a 5975C mass selective detector (MSD, Agilent Technologies, Germany). The gas chromatograph was equipped with an HP-5 MS column (30 × 0.25 mm ID, 0.25 µm film thickness; Agilent Technologies, Inc. USA) with helium as the carrier gas. The combined samples were analyzed with a temperature program of 50 °C for 5 min that increased by 3 °C/min to 320 °C with a final hold time of 10 min. Derivatized samples were analyzed with a temperature program of 50 °C for 5 min, increased by 5 °C/min to 320 °C with a final hold time of 10 min. To compare the chemical composition between samples, relative peak areas were calculated for each of the 20 male, 30 female and web silk extracts separately by standardizing the total peak area of each extract to 100%. Values given in Table 1 are means ± standard deviation (SD). GC on chiral phases was performed using BetaDex™ 225 (Sigma, 30.0 m × 0.25 mm) or Hydrodex β-6TBDM (Machery and Nagel, 30.0 m × 0.25 mm) columns with a flow of 1.5 mL min^−1^ hydrogen as the carrier gas and a flame ionization detector. Individual time programs are given in the respective experimental descriptions and Figs. 7 and S5.

### Discriminant analyses

Individual chemical profiles of 20 male and 29 female cuticles, as well as 22 female webs were compared by discriminant analyses (DA) in SPSS 27.0. One female and eight silk extracts were excluded from the analyses, as only a few peaks were detected in their GC profiles. Only peaks representing > 1% of the mean total peak area in all three sample types were considered (seven peaks in total: *I* 2700, 2900, 3061, 3273, 3380, 3476, 3575 in Table 1). Because relative peak areas constitute compositional data, they were transformed to log-contrasts before statistical analyses (Aitchison 2003). Separate DAs were conducted using all seven peaks > 1%, only wax esters (four peaks), and only hydrocarbons (three peaks), respectively. Relative areas of peaks entering a given DA were again calculated by standardizing their total peak area to 100%.

### Microreactions of Extracts

**Fig. 1:**
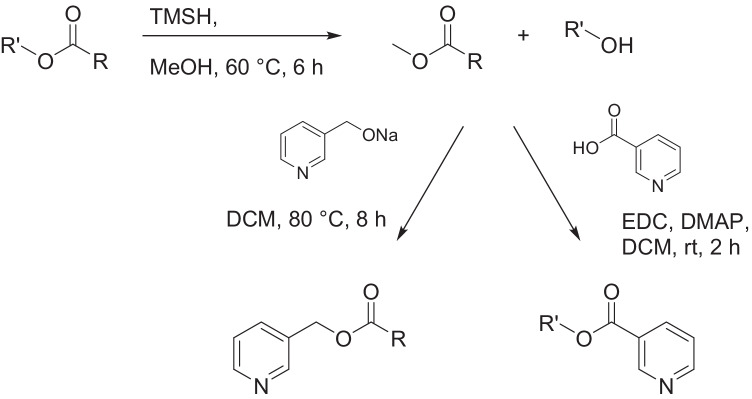
Microreactions performed with *A. bruennichi* extracts. Transesterification with TMSH transformed the wax esters into methyl esters that were again transesterified with 3-pyridinylmethanol or transformed into nicotinates with nicotinic acid

The extracts were derivatized in microreactions to obtain more structural information about methyl-branch positions of the acid and alcohol components of the long chain esters. Extracts were transesterified with trimethylsulfonium hydroxide (TMSH) (Müller et al. 1990) to form the corresponding methyl esters and free alcohols (Fig. 1). Subsequent transesterification of the methyl esters with sodium  3-pyridinylmethoxide to form the corresponding 3-pyridinylmethyl esters was performed (Harvey 1982) as well as esterification of the free alcohols with nicotinic acid to form the corresponding esters (Vetter and Meister 1981; Harvey 1991).

#### Transesterification with Trimethylsulfonium Hydroxide

Trimethylsulfonium hydroxide (TMSH, 100 µl, 0.25 m in methanol) was added to the natural sample (in 20 µl dichloromethane) in a GC-vial (2 mL). The reaction mixture was placed in a heating block at 90 °C for 6 h and regularly shaken vigorously. The solvents and reagents were removed with a stream of nitrogen and the residue was dissolved in DCM (20 µl) (Müller et al. 1990).

#### Transesterification with 3-Pyridinylmethanol

3-Pyridinylmethanol (50 µl) was added to freshly cut sodium (0.2 mg) in a GC-vial (2 mL). The reaction mixture was heated to 80 °C in a heating block until the sodium had dissolved. Sodium 3-pyridinylmethoxide (2% in 3-pyridinylmethanol) was obtained as a syrupy yellow liquid. The freshly prepared sodium 3-pyridinylmethoxide (2 drops, 2% in 3-pyridinylmethanol) was added to the methyl ester sample (20 µl in dichloromethane) in a GC-vial (2 mL). The reaction was placed in a heating block at 80 °C for 3 h and regularly shaken vigorously. Methanol (200 µl) and water (3 drops) were added to the solution and the mixture was extracted with pentane (3 × 200 µl). The pentane phases were combined, the solvent was removed under a stream of nitrogen, and the residue was dissolved in DCM (20 µl).

#### Preparation of Nicotinates

DCM (50 µl), nicotinic acid (1 mg), 1-ethyl-3-(3-dimethylaminopropyl)carbodiimide (EDC, 1 mg) and 4-dimethylaminopyridine (DMAP, catalytic amount) were added to the sample of the methyl esters and free alcohols (50 µl in dichloromethane) in a GC-vial (2 mL). The reaction mixture was kept at room temperature for 2 h and regularly shaken vigorously. The solvents were removed in a stream of nitrogen and the residue was extracted with pentane (3 × 100 µl). The combined pentane phases were again concentrated in a stream of nitrogen and the residue was dissolved in DCM (50 µl).

### General Experimental Procedures

All reactions were performed in oven-dried glassware under a nitrogen atmosphere. Solvents were dried according to standard procedures. Column chromatography was done with silica 60 (0.063–0.200 mm, 70–230 mesh ASTM) and thin layer chromatography (TLC) with Polygram® SIL G/UV silica 60 plates, 0.20 mm thickness. Compounds were visualized with potassium permanganate solution. NMR spectra were recorded either on Avance III HD 300 N (^1^H-NMR: 300 MHz, ^13^C-NMR: 76 MHz), DRX 400 (^1^H-NMR: 400 MHz, ^13^C-NMR: 101 MHz), AVII 400 (^1^H-NMR: 400 MHz, ^13^C-NMR: 101 MHz), or AVII 600 (^1^H-NMR: 600 MHz) instruments. Data are reported as follows: chemical shifts, multiplicity (s = singlet, d = doublet, t = triplet, q = quartet, m = multiplet), coupling constants (Hz). IR spectra were measured on a Bruker Tensor 27 (diamond-ATR). Mass spectra were recorded with a combination of an Agilent Technologies 5977B gas chromatograph connected to an Agilent Technologies 8860 Series MSD. The enantiomeric excess of chiral compounds was determined by GC on BetaDex™ 225 or Hydrodex β-6TBDM [(30.0 m × 0.25 mm) phases, operated with H_2_ as carrier gas with a flow of 1.5 ml min. Optical rotations were determined with an MCP 150 polarimeter (Anton Paar) with a cell length of 10 cm (*c* given in mg/mL).

#### Preparation of *S*-Ethyl (*S*)-4-((*tert*-Butyldiphenylsilyl)oxy)-3-methylbutanethioate (2)

The synthesis was performed according to Horst et al. (2007) using (*R*)-1-[(*S*_P_)-2-(diphenylphosphino)ferrocenyl]ethyldicyclohexylphosphine (Josiphos, **11**, 0.08 mmol, 50 mg, 0.012 eq.) and CuBr·SMe_2_ (0.065 mmol, 13 mg, 0.01 eq.), methyl *tert*-butyl ether (MTBE, 50 mL), MeMgBr (7.78 mmol, solution in diethyl ether), and *S*-ethyl (*E*)-4-((*tert*-butyldiphenylsilyl)oxy)but-2-enethioate (6.48 mmol, 2493 mg, 1 eq.) to afford **2** as a colorless oil (2199 mg, 85%). ${\left[\alpha \right]}_{\text{D}}^{20}$ **=**  − 4.60 (10 mg/mL; CH_2_Cl_2_). FT-IR:* ν* / cm^−1^ = 2960, 2895, 2859, 1688, 1466, 1427, 1389, 1261, 1150, 1108, 1040, 1005, 941, 821, 802, 763, 741, 703, 615. ^1^H-NMR: (400 MHz, CDCl_3_) *δ* / ppm = 7.71–7.66 (m, 4H), 7.47–7.37 (m, 6H), 3.53 (ddd, *J* = 16.2, 9.9, 5.7 Hz, 2H), 2.93–2.82 (m, 3H), 2.40 (dd, *J* = 14.4, 8.4 Hz, 1H), 2.36–2.25 (m, 1H), 1.26 (t, *J* = 7.4 Hz, 3H), 1.08 (d, *J* = 2.8 Hz, 9H), 0.98 (d, *J* = 6.6 Hz, 3H). ^13^C-NMR, DEPT**:** (101 MHz, CDCl_3_) *δ* / ppm = 199.2 (C = O), 135.7 (CH_Ar_), 135.7 (CH_Ar_), 133.8 (CH_Ar_), 133.8 (C_Ar_), 129.7 (CH_Ar_), 127.7 (CH_Ar_), 68.0 (CH_2_), 47.7 (CH_2_), 33.9 (CH), 27.0 (CH_3_), 23.4 (CH_2_), 19.4 (C), 16.6 (CH_3_), 14.9 (CH_3_). EI-MS (70 eV): *m/z* = 344 (23), 343 ([*M* − ^t^Bu]^+^, 76), 244 (26), 243 (100), 197 (13), 183 (39), 181 (22), 135 (25), 137 (23), 105 (16). The enantiomeric excess (ee) was determined by cleavage of the silyl ether using tetrabutylammonium fluoride (TBAF) (Horst et al. 2007). The resulting lactone was analyzed by GC on a chiral phase BetaDex™ 225 (30.0 m × 0.25 mm, initial temp. 50 °C then 10 °C min^−1^ to final temp. 160 °C, Fig. S5). Retention time, 13.75 min (minor), 13.85 (major) showed 94% ee (Lit.: 98% ee Horst et al. 2007).

#### Preparation of *S*-Ethyl (*2E,5S*,*2E*)-6-((*tert*-Butyldiphenylsilyl)oxy)-5-methylhex-2-enethioate (3)

The synthesis was performed as described by Horst et al. (2007) using Et_3_SiH (36.69 mmol, 4266 mg, 3 eq.), **2** (12.23 mmol, 4900 mg, 1 eq.) and 10% Pd/C (5 mol%, 650 mg) in CH_2_Cl_2_ (20 mL) to afford the crude aldehyde that underwent a Wittig reaction with *S*-ethyl 2-(triphenyl-λ^5^-phosphaneylidene)ethanethioate (3.221 mmol, 1174 mg) in CH_2_Cl_2_ (40 mL) to afford **3** as a colorless oil (2010 mg, 39% over two steps). ${\text{[}\alpha \text{]}}_{\text{D}}^{20}$ =  − 6.2 (10 mg/mL; CHCl_3_). ^1^H-NMR: (300 MHz, CDCl_3_) *δ* / ppm = 7.70–7.60 (m, 4H), 7.46–7.33 (m, 6H), 6.93–6.79 (m, 1H), 6.11 (d, *J* = 15.5 Hz, 1H), 3.49 (ddd, *J* = 16.3, 10.0, 5.9 Hz, 2H), 2.94 (q, *J* = 7.4 Hz, 2H), 2.50–2.36 (m, 1H), 2.04 (ddd, *J* = 15.4, 8.4, 7.3 Hz, 1H), 1.94–1.77 (m, 1H), 1.28 (dd, *J* = 7.7, 7.2 Hz, 3H), 1.06 (s, 9H), 0.91 (d, *J* = 6.8 Hz, 3H). ^13^C-NMR, DEPT: (76 MHz, CDCl_3_) *δ* / ppm = 190.1 (C), 144.0 (CH), 135.7 (CH), 135.7 (CH), 133.9 (CH), 133.8 (CH), 130.1, 129.8 (CH), 127.8 (CH), 68.2 (CH_2_), 36.1 (CH_2_), 35.6 (CH), 27.0 (CH_3_), 23.2 (CH_2_), 19.4 (C), 16.6 (CH_3_), 15.0 (CH_3_). EI-MS (70 eV): *m/z*** = **283 ([*M* − ^t^Bu]^+^, 31), 200 (19), 199 (100), 181 (24), 175 (16), 139 (38), 105 (18), 83 (23), 77 (19), 41 (16).

#### Preparation of *S*-Ethyl (3*R*,5*S*)-6-((*tert*-Butyldiphenylsilyl)oxy)-3,5-dimethylhexanethioate (4)

The synthesis was performed according to Horst et al. (2007) using **11** (0.12 mmol, 75 mg, 0.012 eq.), CuBr·SMe_2_ (0.097 mmol, 20 mg, 0.01 eq.) dissolved in MTBE (65 mL), MeMgBr (11.64 mmol, solution in diethyl ether), and **3** (9.70 mmol, 3730 mg, 1 eq.) to afford **4** as a colorless oil (3533 mg, 82%). ${\text{[}\alpha \text{]}}_{\text{D}}^{20}$ =  − 4.3 (10 mg/mL; CHCl_3_). FT-IR:* ν* / cm^−1^ = 2959, 2930, 2860, 1689, 1463, 1427, 1384, 1262, 1108, 1083, 1002, 821, 741, 702, 615. ^1^H-NMR: (300 MHz, CDCl_3_) *δ* / ppm = 7.72–7.66 (m, 4H), 7.47–7.36 (m, 6H), 3.48 (ddd, *J* = 24.5, 9.8, 5.9 Hz, 2H), 2.88 (q, *J* = 7.5 Hz, 2H), 2.53 (dd, *J* = 14.3, 5.0 Hz, 1H), 2.26 (dd, *J* = 14.3, 8.7 Hz, 1H), 2.18–2.02 (m, 1H), 1.81–1.65 (m, 1H), 1.48–1.35 (m, 1H), 1.30–1.21 (m, 3H), 1.12–1.05 (m, 9H), 1.05–0.98 (m, 1H), 0.93 (dt, *J* = 6.7, 4.5 Hz, 6H). ^13^C-NMR, DEPT: (76 MHz, CDCl_3_) *δ* / ppm = 199.3 (C = O), 135.8 (CH_Ar_), 134.1 (C_Ar_), 134.1 (C_Ar_), 129.7 (CH_Ar_), 127.7 (CH_Ar_), 68.9 (CH_2_), 51.3 (CH_2_), 40.9 (CH_2_), 33.3 (CH), 28.8 (CH), 27.0 (CH_3_), 23.4 (CH_2_), 20.4 (CH_3_), 19.4 (C_q_), 17.6 (CH_3_), 14.9 (CH_3_). EI-MS (70 eV): *m/z* = 386 (30), 385 ([*M* − ^t^Bu]^+^, 100), 323 (31), 243 (44), 199 (92), 183 (50), 181 (35), 135 (41), 83 (35), 55 (36).

#### Preparation of (3*R*,5*S*)-6-((*tert*-Butyldiphenylsilyl)oxy)-3,5-dimethylhexan-1-ol (5)

To a solution of **4** (0.90 mmol, 400 mg, 1 eq.) in CH_2_Cl_2_ (10 mL) was added diisobutylaluminium hydride (1.17 mmol, 1.3 eq., solution in cyclohexane) at –50 °C under a nitrogen atmosphere. After stirring for 17 h the reaction mixture was allowed to warm up to room temperature. The reaction was quenched by addition of saturated Rochelle solution (10 mL) and stirred for 30 min at room temperature. The phases were separated and the aqueous phase was extracted three times with CH_2_Cl_2_ (30 mL). The combined organic phases were dried over Na_2_SO_4_ and the solvent removed under reduced pressure to yield the crude aldehyde. The reduction procedure was repeated and the obtained residue was purified by column chromatography (pentane/diethyl ether; 1:1) to afford alcohol **13** as a slightly yellow oil (267 mg, 77%). In contrast to the published procedure (Horst et al. 2007) a two-step reduction proved to be necessary to obtain good yields. ${\left[\mathrm{\alpha }\right]}_{\text{D}}^{20}$ =  − 3.7 (10 mg/mL; CHCl_3_). FT-IR: *ν* / cm^−1^ = 2928, 2859, 1466, 1428, 1385, 1107, 1007, 821, 739, 700, 614. ^1^H-NMR: (300 MHz, CDCl_3_) *δ* / ppm = 7.69 (m, 4H), 7.48–7.36 (m, 6H), 3.74–3.58 (m, 2H), 3.57–3.40 (m, 2H), 1.86–1.68 (d, *J* = 6.6 Hz, 1H), 1.68–1.52 (m, 2H), 1.49–1.22 (m, 3H). ^13^C-NMR, DEPT: (76 MHz, CDCl_3_) *δ* / ppm = 135.8 (CH), 135.8 (C), 134.2 (CH), 129.6 (CH), 127.7 (CH), 68.9 (CH_2_), 61.2 (CH_2_), 41.3 (CH_2_), 39.9 (CH_2_), 33.2 (CH_3_), 27.1 (CH_3_), 27.0 (CH_3_), 20.4 (CH_3_), 19.4 (C_q_), 17.8 (CH_3_). EI-MS (70 eV): *m/z* = 327 ([*M* − ^t^Bu]^+^, 2), 200 (11), 199 (63), 181 (14), 139 (11), 135 (10), 111 (37), 69 (100), 57 (10), 55 (39), 41 (19).

#### Preparation of (3R,5S)-6-((*tert*-Butyldiphenylsilyl)oxy)-3,5-dimethylhexyl 4-Methylbenzenesulfonate (6)

Tosyl chloride (1.35 mmol, 257 mg, 2 eq.) was added to a solution of **5** (0.67 mmol, 259 mg, 1 eq.) and pyridine (1.35 mmol, 109 µL, 2 eq.) in CH_2_Cl_2_ (5 mL). The reaction mixture was stirred at room temperature for 19 h under a nitrogen atmosphere. The solvent was removed under reduced pressure and the resulting residue was purified by column chromatography (pentane/diethyl ether; 20:1) to afford **6** as a colorless oil (289 mg, 80%). ${\left[\mathrm{\alpha }\right]}_{\mathrm{D}}^{20}$ =  − 4.2 (10 mg/mL; CHCl_3_). FT-IR:* ν* / cm^−1^ = 2955, 2923, 2854, 1463, 1428, 1389, 1377, 1362, 1110, 1007, 999, 824, 795, 738, 700, 671, 665, 614, 528. ^1^H-NMR: (300 MHz, CDCl_3_) *δ* / ppm = 7.81–7.75 (m, 2H), 7.70–7.63 (m, 4H), 7.48–7.27 (m, 8H), 4.12–3.96 (m, 2H), 3.42 (ddd, *J* = 16.1, 9.8, 5.9 Hz, 2H), 2.43 (s, 3H), 1.77–1.44 (m, 3H), 1.32 (ddt, *J* = 13.5, 10.8, 6.6 Hz, 3H), 1.05 (s, 9H), 0.99–0.81 (m, 4H), 0.77 (d, *J* = 6.5 Hz, 3H). ^13^C-NMR, DEPT: (76 MHz, CDCl_3_) *δ* / ppm = 144.7 (C_Ar_), 135.7 (CH_Ar_), 134.1 (C_Ar_), 133.4 (C_Ar_), 129.9 (CH_Ar_), 129.7 (CH_Ar_), 128.0 (CH_Ar_), 127.7 (CH_Ar_), 69.2 (CH_2_), 68.8 (CH_2_), 41.1 (CH_2_), 35.7 (CH_2_), 33.1 (CH), 27.0 (CH_3_), 27.0 (CH), 21.7 (CH_3_), 19.9 (C_q_), 19.4 (CH_3_), 17.7 (CH_3_). EI-MS (70 eV): *m/z* = 353 (35), 293 (66), 199 (52), 181 (20), 135 (21), 111 (58), 69 (100), 91 (51), (48), 41 (28).

#### Preparation of (2*S*,4*S*)-*tert*-Butyl(2,4-dimethylheptadecyl)oxydiphenylsilane (7)

1-Bromoundecane was added (2.13 mmol, 500 mg) to a mixture of magnesium turnings (2.55 mmol, 62 mg) in THF (10 mL). The reaction mixture was heated to reflux for 30 min, then cooled to room temperature. Compound **6** (0.51 mmol, 273 mg, 1 eq.) and CuBr·SMe_2_ (0.10 mmol, 21 mg, 0.2 eq.) were dissolved in THF (6 mL) under a nitrogen atmosphere. The freshly prepared Grignard solution was added at 0 °C. After warming to room temperature, the reaction mixture was stirred for 1 h. After quenching with saturated NH_4_Cl solution (6 mL) the phases were separated and the aqueous phase was extracted three times with diethyl ether (30 mL). The combined organic phases were dried over Na_2_SO_4_ and the solvent was removed under reduced pressure. The residue was purified by column chromatography (pentane/diethyl ether; 20:1) to afford **7** as a colorless oil (113 mg, 42%). ${\left[\mathrm{\alpha }\right]}_{\mathrm{D}}^{20}$ =  − 3.3 (10 mg/mL; CHCl_3_). FT-IR:* ν* / cm^−1^ = 3067, 2957, 2929, 2860, 2323, 1596, 1465, 1429, 1361, 1300, 1258, 1213, 1180, 1103, 945, 892, 816, 744, 701, 663, 614, 578, 555. ^1^H-NMR: (300 MHz, CDCl_3_) *δ* / ppm = 7.73–7.63 (m, 4H), 7.50–7.31 (m, 6H), 3.47 (ddd, *J* = 16.2, 9.8, 5.9 Hz, 2H), 1.74 (d, *J* = 6.7 Hz, 1H), 1.50–1.19 (m, 25H), 1.10–1.03 (m, 9H), 0.96–0.85 (m, 7H), 0.82 (d, *J* = 6.4 Hz, 3H). ^13^C-NMR, DEPT: (76 MHz, CDCl_3_) *δ* / ppm = 135.8 (CH_Ar_), 134.3 (C_Ar_), 129.6 (CH_Ar_), 127.7 (CH_Ar_), 69.1 (CH_2_), 41.3 (CH_2_), 37.0 (CH_2_), 33.3 (CH), 32.1 (CH_2_), 30.2 (CH_2_), 30.2 (CH), 29.9 (CH_2_), 29.9 (CH_2_), 29.8 (CH_2_), 29.5 (CH_2_), 27.0 (CH_3_), 27.0 (CH_2_), 22.9 (CH_2_), 20.5 (CH_3_), 19.5 (C_q_), 17.9 (CH_3_), 14.3 (CH_3_). EI-MS (70 eV): *m/z* = 465 (34), 200 (19), 199 (100), 97 (23), 83 (25), 83 (19), 69 (28), 57 (40), 55 (20), 43 (37).

#### Preparation of (2*S*,4S)-2,4-Dimethylheptadecan-1-ol (8)

TBAF (0.57 mmol, 574 µL, 3 eq.) was added to a solution of **7** (0.19 mmol, 100 mg, 1 eq.) in THF (5 mL). The reaction mixture was stirred at room temperature for 24 h under a nitrogen atmosphere. The solvent was removed under reduced pressure and the residue was purified by column chromatography (pentane/diethyl ether; 20:1) to afford **8** as a colorless oil with traces of siloxanes as impurities (21 mg, crude). ${\left[\mathrm{\alpha }\right]}_{\mathrm{D}}^{20}$ =  − 37.6 (10 mg/mL; CHCl_3_). FT-IR:* ν* / cm^−1^ = 3341, 2955, 2922, 2853, 1462, 1377, 1112, 1036, 987, 865, 821, 704, 606. ^1^H-NMR: (600 MHz, CDCl_3_) δ 3.49–3.27 (m, 2H), 1.70–1.60 (m, 1H), 1.46–1.37 (m, 1H), 1.27–1.10 (m, 26H), 1.09–0.91 (m, 2H), 0.90–0.75 (m, 10H). ^13^C-NMR, DEPT: (151 MHz, CDCl3) *δ* / ppm = 68.2 (CH_2_), 40.9 (CH_2_), 36.5 (CH_2_), 32.9 (CH), 31.7 (CH_2_), 29.9 (CH), 29.8 (CH_2_), 29.5 (CH_2_), 29.5 (CH_2_), 29.5 (CH_2_), 29.2 (CH_2_), 26.7 (CH_2_), 22.5 (CH_2_), 20.2 (CH_3_), 17.1 (CH_3_), 13.9 (CH_3_). EI-MS (70 eV): *m/z* = 283 (< 1), 266 (< 1), 224 (10), 209 (6), 196 (7), 168 (7), 111 (22), 97 (31), 83 (100), 71 (44), 70 (39), 69 (52), 57 (97), 56 (60), 55 (85), 43 (81), 41 (61).

#### Preparation of (2*S*,4*S*)-2,4-Dimethylheptadecanoic Acid (9)

Under a nitrogen atmosphere, RuCl_3_ (0.014 mmol, 3 mg, 0.3 eq.) and NaIO_4_ (0.281 mmol, 60 mg, 5 eq.) were added to a mixture of crude **8** (0.056 mmol, 16 mg, 1 eq.), H_2_O (1.2 mL), CH_3_CN (1.2 mL) and CCl_4_ (2.4 mL). The mixture was stirred at room temperature for 3.5 h. After addition of CH_2_Cl_2_ (4 mL) and H_2_O (1 mL) the phases were separated and the aqueous phase was extracted three times with CH_2_Cl_2_ (10 mL). The combined organic phases were dried over Na_2_SO_4_. The solvent was removed under reduced pressure and the residue was purified by column chromatography (pentane/ethyl acetate/acetic acid; 90:10:1) to afford acid **9** as a colorless oil (11 mg, 66%). ${\left[\mathrm{\alpha }\right]}_{\mathrm{D}}^{20}$ =  + 7.0 (10 mg/mL; CHCl_3_). FT-IR: *ν* / cm^−1^ = 2956, 2923, 2853, 1815, 1707, 1464, 1416, 1379, 1290, 1235, 1091, 1018, 947, 810, 722, 529. ^1^H-NMR: (600 MHz, CDCl_3_) *δ* / ppm = 9.58 (d, *J* = 2.5 Hz, 1H), 2.67–2.51 (m, 1H), 1.80–1.66 (m, 1H), 1.53–1.41 (m, 1H), 1.40–1.02 (m, 31H), 0.95–0.79 (m, 7H). ^13^C-NMR, DEPT: (151 MHz, CDCl_3_) *δ* / ppm = 172.7 (C), 41.4 (CH_2_), 37.2 (CH_2_), 32.1 (CH_2_), 30.9 (CH_2_), 30.1 (CH), 29.9 (CH_2_), 29.5 (CH_2_), 26.9 (CH_2_), 22.9 (CH_2_), 19.7 (CH_3_), 18.0 (CH_3_), 14.27 (CH_3_). A small portion was converted into the respective methyl ester by treatment with trimethylsilyldiazomethane. EI-MS (70 eV): *m/z* = 312 ([*M*]^+^, 3), 241 (12), 129 (7), 101 (59), 88 (100), 71 (7), 69 (12), 57 (13), 55 (12), 43 (12), 41 (10).

#### Preparation of Tetradecyl (2*S*,4*S*)-2,4-Dimethylheptadecanoate (10)

1-Tetradecanol (0.007 mmol, 0.53 mg, 1 eq., 1%wt in DCM), dicyclohexylcarbodiimide (0.007 mmol, 1.28 mg, 1 eq., 1%wt in DCM) and 4-dimethylaminopyridine (0.007 mmol, 1.38 mg, 1 eq., 1%wt in DCM) were added to a solution of (2*S*,4*S*)-2,4-dimethylheptadecanoic acid (**9**) (0.007 mmol, 2 mg, 1 eq.) in DCM (5 mL). After stirring for 20 h at room temperature the solvent was removed under reduced pressure and the residue was purified by column chromatography (pentane/diethyl ether; 100:1) to afford **11** as a colorless oil (2 mg, 58%). ${\left[\mathrm{\alpha }\right]}_{\mathrm{D}}^{20}$ =  + 8.0 (2 mg/mL; CHCl_3_). FT-IR: ν / cm^−1^ = 2923, 2855, 2319, 1736, 1461, 1372, 1172, 673, 603, 565, 546. ^1^H-NMR: (500 MHz, CDCl_3_) *δ* / ppm = 4.11–3.99 (m, 2H), 2.58–2.46 (m, 1H), 1.74–1.66 (m, 1H), 1.65–1.57 (m, 3H), 1.42–1.17 (m, 38H), 1.16–1.05 (m, 5H), 0.91–0.78 (m, 10H). ^13^C-NMR, DEPT: (126 MHz, CDCl_3_) *δ* / ppm = 177.4 (C_q_), 64.4 (CH_2_), 41.8 (CH_2_), 37.7 (CH), 37.3 (CH_2_), 32.1 (CH_2_), 31.0 (CH), 30.1 (CH_2_), 29.9 (CH_2_), 29.8 (CH_2_), 29.8 (CH_2_), 29.7 (CH_2_), 29.5 (CH_2_), 29.4 (CH_2_), 28.9 (CH_2_), 27.0 (CH_2_), 26.1 (CH_2_), 22.9 (CH_2_), 19.7 (CH_3_), 18.2 (CH_3_), 14.3 (CH_3_). EI-MS (70 eV): m/z (%) = 299 (100), 241 (17), 196 (23), 111 (14), 97 (24), 87 (50), 83 (25), 75 (30), 74 (61), 71 (36), 69 (31), 57 (55), 55 (57), 43 (41), 41 (17).

## Results

### Analysis of Spider Extracts by GC/MS

**Fig. 2:**
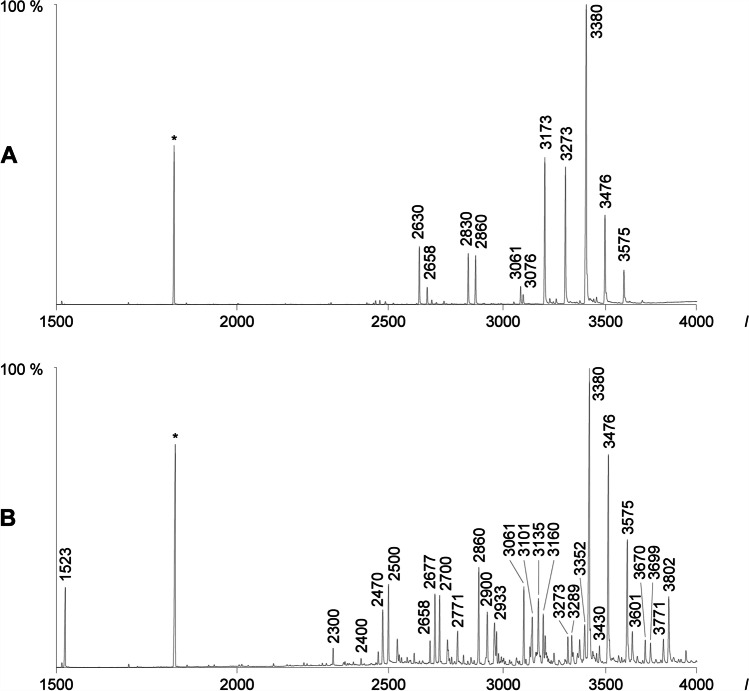
Total ion chromatograms of the combined cuticular extracts from male (A) and female (B) *A. bruennichi*. *Octadecane as internal standard for quantification

Dichloromethane extracts of silk from females and of the cuticle of both sexes of *Argiope bruennichi* were individually analyzed by GC/MS and the average relative proportions of the compounds were determined (Table 1). The samples were then sex-specifically combined to allow identification of minor components by GC/MS (Fig. 2). The female-produced sex pheromone, trimethyl methylcitrate (*I* 1523), as well as 3-octanoyloxy-γ-butyrolactone, a compound of unknown function specific to silk from females (Chinta et al. 2010), were the only compounds eluting early. Besides some hydrocarbons, the dominant cuticular lipids showed mass spectra consistent with wax-type esters (McLafferty and Turecek 1993; Chinta et al. 2016). These spectra are dominated by ions formed in a characteristic McLafferty rearrangement that cleaves the O-alkyl bond and, together with hydrogen transfer, leads to the protonated acid and alkene ions.

**Table 1:** LIST OF COMPOUNDS DETECTED IN EXTRACTS OF *A*. *BRUENNICHI*. Individual samples of 30 bodies and webs of females, as well as samples from 20 bodies of males, were analyzed. Compounds often coeluted within one peak. The average percentage of each peak within the whole sample is reported. Peaks are separated with lines in the table. Major components of peak groups are marked in bold. The three most abundant compounds of each sample type are also marked in bold in the columns on the right

		Female body		Female web		Male body	
*I*	Substance	mean %	SD	mean %	SD	mean %	SD
1523	Trimethyl methylcitrate	0.84	0.77	0.95	1.17	**—**	
1803	3-Octanoyloxy-γ-butyrolactone	—		3.07	2.83	**—**	
1900	Nonadecane	tr		0.20	0.18	**—**	
1946	Hexadecenoic acid	0.08	0.30	—		**—**	
1959	Hexadecanoic acid	0.08	0.14	1.33	1.65	0.17	0.12
1992	Ethyl hexadecanoate	tr		0.65	0.29	—	
	**Eicosane**						
2090	Nonadecanal	0.10	0.13	tr		—	
2100	Heneicosane	0.10	0.07	1.25	0.44		
2142	Octadecadienoic acid	tr		—		tr	
	Octadecenoic acid						
2160	Octadecanoic acid	tr		0.68	1.02	0.22	0.54
	Ethyl octadecenoate						
2193	Ethyl octadecanoate	0.09	0.06	1.50	0.57	—	
	Docosane						
2209	Unknown	tr		—		—	
2259	2-Methyldocosane	0.07	0.04	0.91	0.47	—	
2276	Tricosene	0.07	0.14	0.07	0.12	—	
2283	Tricosene	tr		—		—	
2300	Tricosane	1.56	1.01	3.00	1.03	0.24	0.18
	**1-Eicosanol**						
2334	9-Methyltricosane	0.07	0.05	0.49	0.30	—	
2339	7-Methyltricosane						
2348	5-Methyltricosane	tr		tr		—	
2371	3-Methyltricosane	tr		0.23	0.18	—	
2400	Tetracosane	0.13	0.08	1.59	0.57	tr	
2459	2-Methyltetracosane	0.15	0.07	0.36	0.25	0.06	0.11
2470	3-Methyltetracosane	tr		0.18	0.18	—	
2474	Pentacosene	tr		—		—	
2483	Pentacosene	tr		—		—	
2500	Pentacosane	3.74	1.71	3.30	1.19	0.99	0.37
2527	11-Methylpentacosane + impurity^1^			—		—	
2531	9-Methylpentacosane + impurity^1^						
2538	7-Methylpentacosane	tr		—		tr	
2547	5-Methylpentacosane	tr		0.09	0.18	—	
2558	2-Methylpentacosane	tr		0.17	0.18	tr	
2571	3-Methylpentacosane	tr		0.28	0.23	—	
2592	Unknown	tr		—			
2600	Hexacosane	0.16	0.06	1.08	0.42	tr	
2628	Unknown	0.09	0.07	0.58	0.51	2.80	1.04
2658	2-Methylhexacosane	0.19	0.11	0.64	0.48	—	
2677	Heptacosene	tr		—		—	
2700	Heptacosane + impurity	3.76	1.32	5.99	2.04	1.13	0.45
2733	7-Methylheptacosane + impurity^1^			—		—	
2748	5-Methylheptacosane	tr		—		—	
2758	Hydrocarbon	tr		0.47	0.59	—	
2771	3-Methylheptacosane	0.12	0.08	0.24	0.35	0.28	0.32
2830	Unknown	0.21	0.17	—		4.13	1.96
2842	Unknown	tr		—		—	
2860	2-Methyloctacosane	1.03	0.60	0.54	0.55	1.46	0.61
2871	3-Methyloctacosane	tr		0.17	0.21	—	
2900	Nonacosane	2.23	0.82	2.55	0.60	1.00	0.40
2905	Unknown	tr		0.30	0.98	—	
2929	15-Methylnonacosane	1.31	0.80	2.08	1.36	0.41	0.38
2933	13-Methylnonacosane						
	11-Methylnonacosane						
	9-Methylnonacosane						
2938	7-Methylnonacosane	0.69	0.39	0.44	0.37	tr	
2948	5-Methylnonacosane	0.17	0.10	0.10	0.20	—	
2955	Hydrocarbon	tr		—		—	
2960	Hydrocarbon	0.09	0.09	tr		0.12	0.25
2966	7,11-Dimethylnonacosane	tr		—		tr	
2971	3-Methylnonacosane	0.11	0.06	2.14	1.62	0.23	0.26
2978	5,9-Dimethylnonacosane	tr		—		0.15	0.19
3000	Triacontane	0.20	0.10	1.83	0.73	—	
3008	Hydrocarbon	tr		0.39	0.38	—	
3030	15-Methyltriacontane	0.51	0.35	1.83	2.72	0.70	0.39
	16-Methyltriacontane						
3042	6-Methyltriacontane	0.08	0.07	0.23	0.28	—	
3061	2-Methyltriacontane	1.82	0.90	1.11	0.65	1.72	0.70
3072	Hydrocarbon	0.10	0.12	0.32	0.36	—	
3076	Tridecyl stearate	—		—		1.59	0.54
	**Dodecyl nonadecanoate**						
3091	2,12-Dimethyltriacontane	0.42	0.38	0.14	0.30	—	
	2,14-Dimethyltriacontane						
3101	2,6-Dimethyltriacontane	1.70	0.88	2.69	0.80	0.48	0.37
	2,8-Dimethyltriacontane						
3114	Hydrocarbon	0.25	0.29	0.35	0.42	—	
3118	Hydrocarbon						
3122	Hydrocarbon	0.16	0.20	0.09	0.36	—	
3129	15-Methylhentriacontane	2.58	1.55	2.69	1.71	—	
3135	13-Methylhentriacontane						
	11-Methylhentriacontane						
	9-Methylhentriacontane						
3139	7-Methylhentriacontane	0.25	0.13	0.27	0.43	—	
3160	Hydrocarbon	0.53	0.40	0.48	0.44	—	
3166	Hydrocarbon	0.26	0.19	—		—	
3173	**Tridecyl *syn*-2,4-dimethylheptadecanoate**	0.29	0.24	0.22	0.41	**14.88**	2.44
	5,15-Dimethylhentriacontane						
3176	Tridecyl *anti*-2,4-dimethylheptadecanoate						
3190	Unknown	0.06	0.09	—		—	
3199	Tridecyl 2,4,8-trimethylheptadecanoate	0.45	0.36	1.72	0.88	0.70	0.39
3217	Tridecyl 2,4,14-trimethylheptadecanoate	—		—		0.21	0.25
	Tetradecyl 2,4,14-trimethylhexadecanoate						
3226	16-Methyldotriacontane	0.43	0.28	1.91	2.00	0.89	0.22
3230	14-Methyldotriacontane						
	**Tridecyl 2,4,16-trimethylheptadecanoate**						
	Tetradecyl 2,4,16-trimethylhexadecanoate						
3256	6-Methyldotriacontane	0.16	0.16	—		—	
3260	2-Methyldotriacontane	0.20	0.12	—		—	
3273	Tetradecyl 2,4-dimethylheptadecanoate	1.19	1.08	1.05	0.88	**16.03**	1.12
	**Tridecyl 2,4-dimethyloctadecanoate**						
	Dodecyl 2,4-dimethylnonadecanoate						
3289	Unknown	0.86	0.58	0.34	0.44	—	
3300	Tritriacontane	0.51	0.26	1.31	0.69	—	
3316	Unknown	0.33	0.33	0.24	0.31	—	
3323	17-Methyltritriacontane	2.45	0.92	2.41	1.12	—	
3329	11-Methyltritriacontane						
3336	9-Methyltritriacontane						
	Unknown						
3330	Tetradecyl 2,4,14-trimethylheptadecanoate	—		—		0.35	0.33
3352	7,11,15-Trimethyltritriacontane	1.36	1.05	0.78	0.75	0.29	0.28
3360	11,21-Dimethyltritriacontane	0.35	0.33	0.13	0.29	—	
3380	**Tridecyl 2,4-dimethylnonadecanoate**	**15.77**	7.93	**10.30**	7.15	**32.94**	2.79
	Tetradecyl 2,4-dimethyloctadecanoate						
3384	Tridecyl 2,4,6-trimethylnonadecanoate	0.72	0.72	0.17	0.39	—	
3397	**Tridecyl 2,4,8-trimethylnonadecanoate**	0.52	0.50	0.93	0.64	0.15	0.31
3405	Tridecyl 2,4,10-trimethylnonadecanoate						
3409	Tridecyl 2,4,12-trimethylnonadecanoate	0.09	0.15	—		—	
3416	**Tetradecyl 2,4,14-trimethyloctadecanoate**	0.33	0.13	—		0.21	0.24
	Tridecyl 2,4,14-trimethylnonadecanoate						
3430	Tetradecyl 2,4,16-trimethylocatadecanoate	0.79	0.27	1.91	1.07	0.75	0.29
	**Tridecyl 2,4,16-trimethylnonadecanoate**						
3445	Tetradecyl trimethyloctadecanoate	tr		—		—	
	Pentadecyl trimethylheptadecanoate						
3476	Tetradecyl *syn*-2,4-dimethylnonadecanoate^1^	**12.89**	4.51	**7.38**	4.00	11.25	1.65
	**Tridecyl *syn*-2,4-dimethylicosanoate**						
	Dodecyl *syn*-2,4-dimethylhenicosanoate						
3481	Tetradecyl *anti*-2,4-dimethylnonadecanoate	0.67	0.64	0.28	0.81	0.22	0.29
	**Tridecyl *anti*-2,4-dimethylicosanoate**						
	Dodecyl *anti*-2,4-dimethylhenicosanoate						
3495	Pentadecyl 2,4,6-trimethyloctadecanoate	0.14	0.18	—		—	
	Tetradecyl 2,4,6-trimethylnonadecanoate						
	**Tridecyl 2,4,6-trimethylicosanoate**						
3500	Wax ester	0.13	0.13	0.71	0.51	—	
3504	Wax ester	tr		—		—	
3528	Tetradecyl 2,4,14-trimethylnonadecanoate	0.96	0.29	1.93	0.96	0.06	0.20
	Pentadecyl 2,4,14-trimethyloctadecanoate						
	Hexadecyl 2,4,14-trimethylheptadecanoate						
3544	**Pentadecyl 2,4,16-trimethyloctadecanoate**	0.33	0.12	—		—	
	Tridecyl 2,4,16-trimethylicosanoate						
3557	Hydrocarbon	1.07	0.88	0.41	0.76	0.29	0.76
3575	**Tridecyl *syn*-2,4-dimethylhenicosanoate**	**11.97**	5.03	**6.51**	4.88	2.74	0.83
	Tetradecyl *syn*-2,4-dimethylicosanoate						
	Pentadecyl *syn*-2,4-dimethylnonadecanoate						
3579	**Tridecyl *anti*-2,4-dimethylhenicosanoate**	0.73	0.75	0.24	0.89	**—**	
	Tetradecyl *anti*-2,4-dimethylicosanoate						
	Pentadecyl *anti*-2,4-dimethylnonadecanoate						
3596	Tridecyl 2,4,6-trimethylhenicosanoate	0.17	0.29	—		**—**	
3601	**Octadecyl 2,4,6-trimethylhexadecanoate**	2.46	1.32	1.43	1.23	**—**	
	Hexadecyl 2,4,6-trimethyloctadecanoate						
3627	Pentadecyl 2,4,16-trimethyloctadecanoate	0.62	0.17	1.35	0.85	**—**	
3656	4-Methyloctadecyl 2,4-dimethylheptadecanoate	0.17	0.16	—		**—**	
	4-Methylheptadecyl 2,4-dimethyloctadecanoate						
	4-Methylhexadecyl 2,4-dimethylheptadecanoate						
3670	Ooctadecyl 2,4-dimethylheptadecanoate	2.49	0.96	1.03	0.87	**—**	
	Heptadecyl 2,4-dimethyloctadecanoate						
	Hexadecyl 2,4-dimethylnonadecanoate						
	Pentadecyl 2,4-dimethylicosanoate						
	**Tetradecyl 2,4-dimethylhenicosanoate**						
	Tridecyl 2,4-dimethyldocosanoate						
3676	Wax ester	0.10	0.20	—		**—**	
3687	Wax ester	tr		—		**—**	
3699	Nonadecyl 2,4,6-trimethylhexadecanoate	1.63	1.01	0.87	0.76	**—**	
	**Octadecyl 2,4,6-trimethylheptadecanoate**						
	Heptadecyl 2,4,6-trimethyloctadecanoate						
3728	Heptadecyl 2,4,14-trimethylheptadecanoate	0.32	0.18	0.93	0.81	**—**	
3755	Unknown	0.43	0.24	—		**—**	
3771	Nonadecyl 2,4-dimethylheptadecanoate	2.57	1.50	0.62	0.83	**—**	
	**Heptadecyl 2,4-dimethylnonadecanoate**						
	Pentadecyl 2,4-dimethylhenicosanoate						
3802	**Icosyl 2,4,6-trimethylhexadecanoate**	5.10	3.28	2.22	1.73	**—**	
	Octadecyl 2,4,6-trimethyloctadecanoate						
3826	**Octadecyl 2,4,14-trimethyloctadecanoate**	0.21	0.25	0.59	0.64	**—**	
	Nonadecyl 2,4,14-trimethylheptadecanoate						
3855	4-Methylnonadecyl 2,4-dimethyloctadecanoate	0.08	0.12	—		**—**	
3869	Icosyl 2,4-dimethylheptadecanoate	0.31	0.27	—		**—**	
	**Nonadecyl 2,4-dimethyloctadecanoate**						
	Octadecyl 2,4-dimethylnonadecanoate						
	Heptadecyl 2,4-dimethylicosanoate						
3897	Octadecyl 2,4,6-trimethyloctadecanoate	0.89	0.70	—		**—**	

^1^The compound co-elutes with impurities and was not included in the calculation.

^2^*Syn*/*anti*-assignments were possible when two peaks with identical mass spectra but different *I* occurred. The synthetic material proved that the *syn*-esters elute before *anti*-esters on the DB-5 GC phase.

### Statistical Analyses

**Fig. 3:**
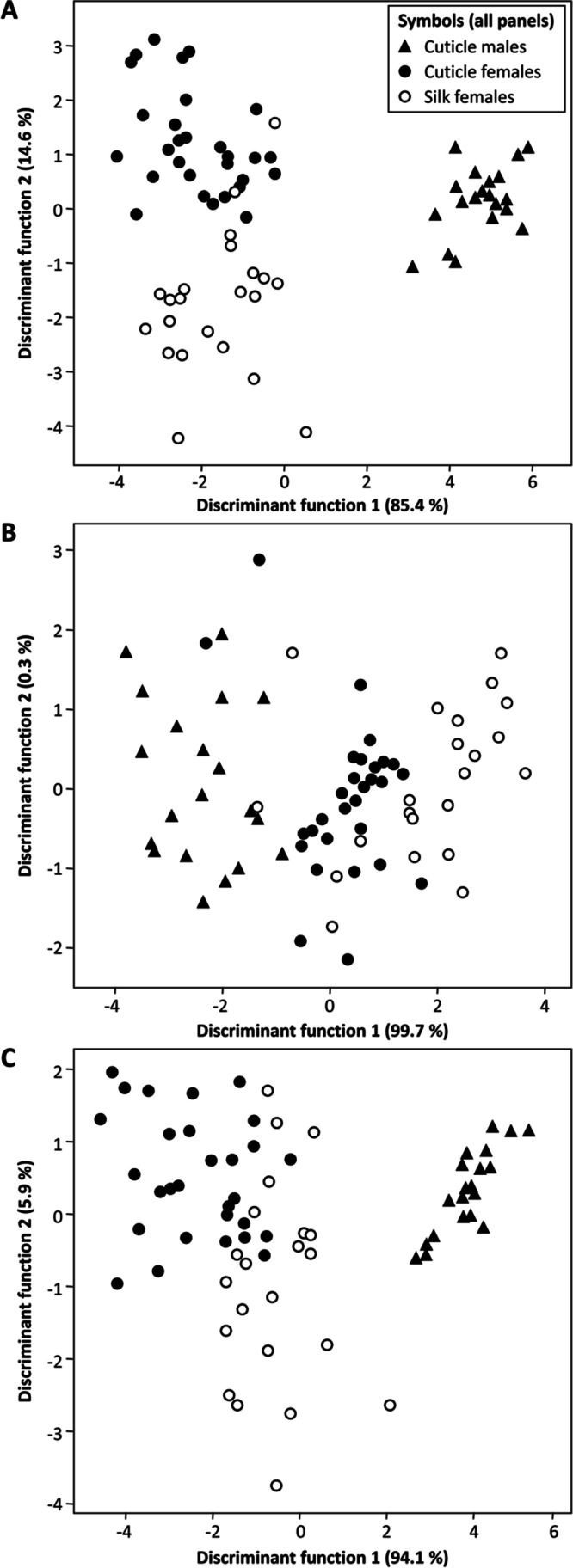
Discriminant analyses of cuticular extracts from males and females, as well as web silk from females, based on all peaks > 1% (A), only hydrocarbons (B), and only wax esters (C). Values in parentheses behind discriminant functions give percentages of explained variance

We then performed discriminant analyses (DAs). To reduce overfitting due to a large influence of minor components, only peaks that were present in more than 1% in all samples were used. These included three hydrocarbons (I 2700, 2900, 3061) and four wax esters (3273, 3380, 3476, 3575). For all seven compounds (Wilk’s lambda = 0.037, Chi2 = 215.9, p < 0.0001, d.f. = 12), the three hydrocarbons (Wilk’s lambda = 0.27, Chi2 = 88.9, p < 0.0001, d.f. = 4), and the four wax esters (Wilk’s lambda = 0.088, Chi2 = 163.0, p < 0.0001, d.f. = 6) the DAs significantly separated the three sample types (Fig. 3). In all analyses, most of the observed variation is explained by the first discriminant function, separating male from female samples, while cuticular and web silk samples from females were mostly separated by the second function and generally grouped closer together.

### Identification of Wax Esters

The wax esters were identified by interpretation of their mass spectra, derivatization procedures, and synthesis. Because many different esters were present, we will illustrate the identification procedure by one example.

**Fig. 4:**
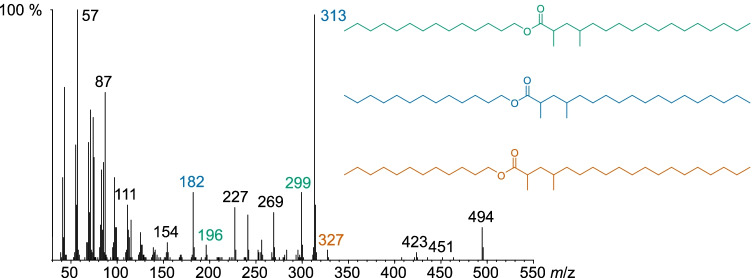
Mass spectrum and structures of 2,4-dimethylalkyl wax ester mixture with *I* 3273

The mass spectrum of the peak with a retention index *I* of 3273 in samples from both males and females (Fig. 4) showed a molecular ion of *m*/*z* 494 and a large peak at *m*/*z* 313, likely the protonated acid ion C_20_H_41_O_2_^+^. The ion *m*/*z* 182 (likely C_13_H_26_^+^) corresponds to the alcohol part. Additionally, smaller peaks at *m*/*z* 299 and 327 were present, along with *m*/*z* 196 and 168. These peak pairs indicate that three esters elute together, consisting of C_19_-acid and C_14_-alcohol, C_20_-acid and C_13_-alcohol, and C_21_-acid and C_12_-alcohol. A characteristic ion at *m*/*z* 74 proved a methyl group at C-2 of the acid (Chinta et al. 2016).

To identify the number and position of methyl groups in the chains, microreactions with the extracts were performed. The natural samples were transesterified with TMSH to the corresponding methyl esters and free alcohols (Müller et al. 1990). The methyl esters allow determination of methyl group positions near the carbonyl group (Ryhage and Stenhagen 1960a). These esters were then again transesterified with sodium 3-pyridinylmethoxide to form the corresponding 3-pyridinylmethyl esters, while the free alcohols were esterified under Steglich conditions with nicotinic acid to the corresponding nicotinates. All these derivatized extracts were analyzed by GC/MS and the data were used to identify the number and position of methyl branches in both acids and alcohols (Fig. 1 and Tables S1 and S2 in the SI).

**Fig. 5:**
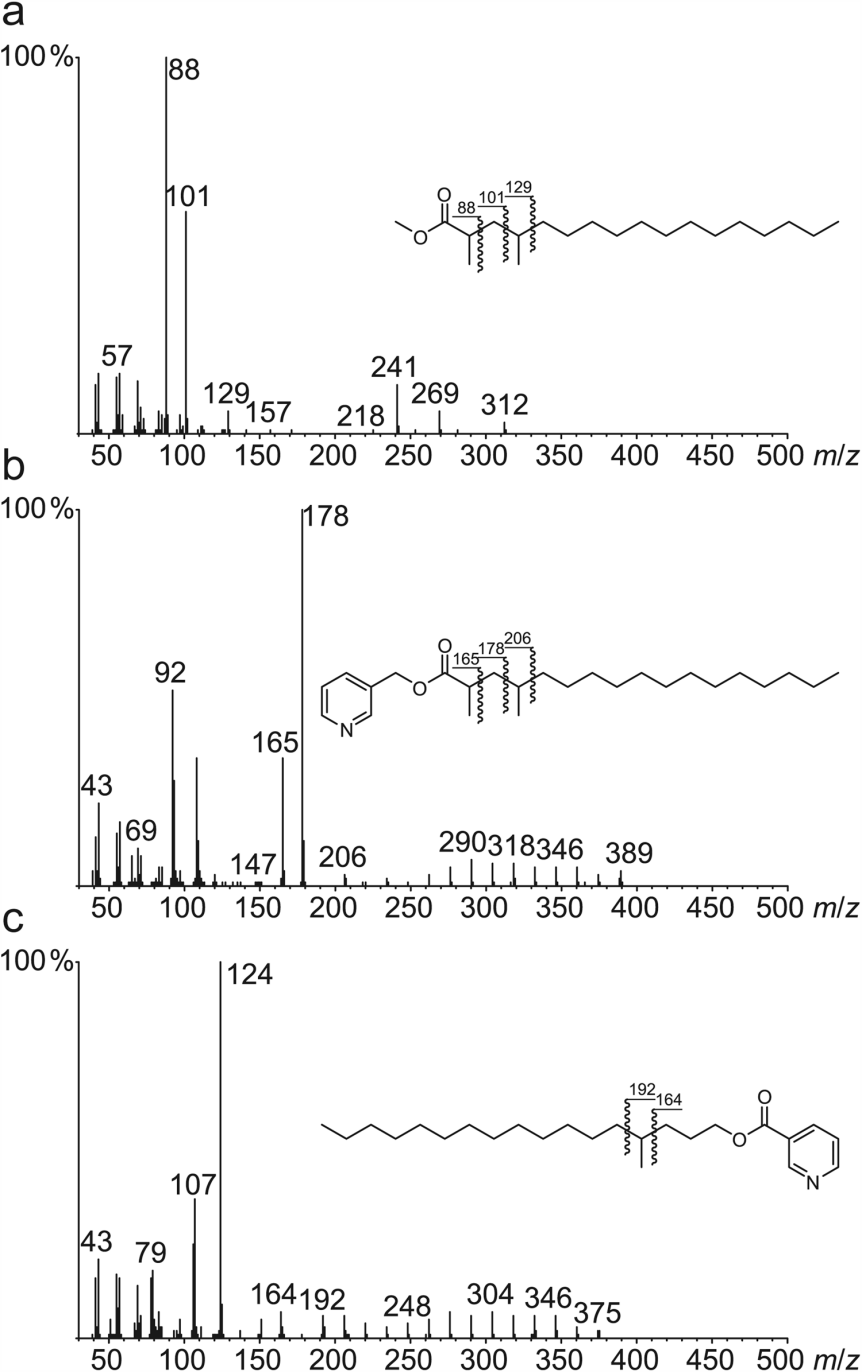
Mass spectra of methyl 2,4-dimethylheptadecanoate (a), 3-pyridinylmethyl 2,4-dimethylheptadecanoate (b), and 4-methylheptadecyl nicotinate (c), derived from wax esters

Methyl esters display characteristic fragment ions formed by β-cleavage and McLafferty rearrangement. Because C-2 substituents are included into the rearrangement, unsubstituted methyl esters have a base peak of *m*/*z* 74 while the base peak is *m*/*z* 88 for 2-methyl-substituted methyl esters (Ryhage and Stenhagen 1960a), as is the case for a major component of both derivatized samples (Fig. 5a). Ryhage and Stenhagen (1960b) showed that polymethyl substituted methyl esters display characteristic ratios of fragment ions. The base peak at *m*/*z* 88 together with higher intensities of *m*/*z* 101 and 129 as well as the ester specific loss of C-2/C-3 + H (*m*/*z* 269, [M − 43]^+^) and C-2/C-3/C-4 + H (*m*/*z* 241, [M − 71]^+^) indicate a 2,4-dimethyl substitution pattern (Ryhage and Stenhagen 1960b). However, additional methyl groups along the chain cannot be reliably deduced from the methyl ester spectra. Therefore, the methyl esters were transformed into 3-pyridinylmethyl esters. Such esters display a regular fragmentation pattern of successive losses of CH_2_ units, with branches resulting in gaps in the pattern and increased intensity in the fragments next to the branching position because of the stability of the resulting secondary radical (Harvey 1991).

The mass spectrum of this derivative is shown in Fig. 5b. The ion *m*/*z* 165 together with the base peak *m*/*z* 178 and the gap in the regular fragmentation pattern between *m*/*z* 178 and *m*/*z* 206 support a 2,4-dimethylheptadecanoate structure of the acid part of the natural esters. This identification was verified using synthetic 2,4-dimethylheptadecanoic acid (**9**) (see below). Esterification of **9** under the same conditions as performed with the natural samples led to methyl 2,4-dimethylheptadecanoate and 3-pyridinylmethyl 2,4-dimethylheptadecanoate and confirmed our identification (mass spectra see SI, Figs. S1 and S2).

Most of the naturally occurring esters were identified using the described procedures. As can be seen from Table 1, the 2,4-dimethyl motif dominates within the natural esters. In addition, small amounts of 2,4,6-trimethylalkanoate esters and several other 2,4,x-trimethylalkanoates esters were identified using the approach described.

The alcohol parts of the esters were identified as *n*-alkan-1-ols using 3-pyridinecarboxylate derivatives (nicotinates), that display a regular fragmentation pattern of successive losses of CH_2_ units. In addition, the nicotinates obtained from the females showed small amounts of 4-methyl alcohols. A typical mass spectrum is shown in Fig. 5c, displaying the branch-indicating gap between *m/z* 164 and 192.

With these results in hand, we were able to identify most of the natural wax esters (Table 1). Cuticular and silk extracts from females contained acids with 17–24 carbons, with mostly 2,4-dimethyl substitution, as well as smaller amounts of 2,4,6-trimethyl substitution and various other 2,4,x-trimethyl substitution patterns. The alcohol portions were mainly *n*-alkan-1-ols, along with small amounts of 4-methyl alcohols, with chain lengths between 13–24 carbons. Extracts from males contained the same acids, although the shorter acids were more prominent. The alcohols were identified as *n*-alkan-1-ols with chain lengths between 12–15 carbons. The identification of *n*-alkyl 2,4-dimethylalkanoates was verified through the synthesis of tetradecyl 2,4-dimethylheptadecanoate (**10**), as described in the following section.

The characterization of the acid and alcohol components was used to assign proposed structures to the wax esters of the natural samples basing on their mass spectra and *I* (Table 1). The largest signals in the derivatized, as well as the natural samples, were from 2,4-dimethylalkanoates, indicating these to be the major lipid components. The remaining wax esters were assumed to have similar relative retention indices as the derivatives (Schulz 2001) and were identified as the respective 2,4,x-trimethylalkanoates. In the samples from males, small amounts of unbranched esters were present, while in samples from females, small amounts of 4-methylalkyl 2,4-dimethylalkanoates were found.

### Synthesis of a Model Wax Ester

**Fig. 6:**
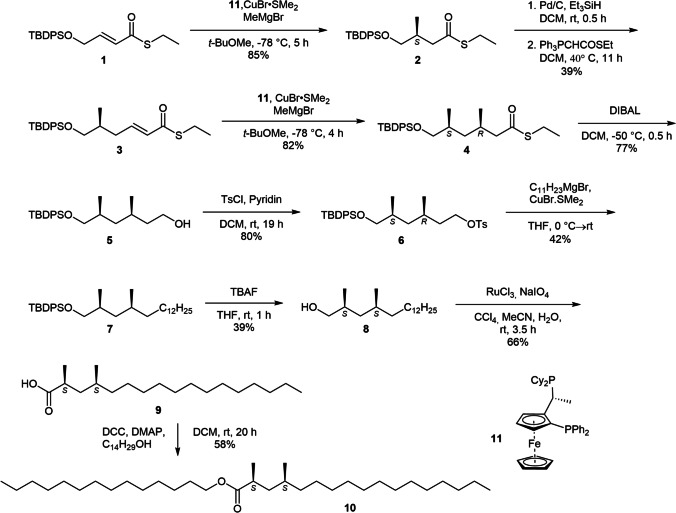
Synthesis of tetradecyl (2*S*,4*S*)-2,4-dimethylheptadecanoate (**10**). Please note that the configurational prefixes change during the synthesis due to the CIP-rules

To confirm our identification, a representative ester, tetradecyl (2*S*,4*S*)-2,4-dimethylheptadecanoate (**10**) was synthesized using the strategy of Feringa et al. for the stereoselective construction of bishomolog methyl-branched acids (Fig. 6) (Des Mazery et al. 2005; Horst et al. 2007; Ruiz et al. 2007). The synthetic material was also used to determine the configuration of the natural esters. The synthesis uses an enantioselective conjugate addition of methylmagnesium bromide to α,β-unsaturated thioesters, using the enantiomers of Josiphos (**11**) as ligands in the key steps. The configuration of each stereogenic center can thus be controlled (Horst et al. 2007).

Thus, the first stereogenic center was introduced into building block **1** with an *S*/*R* ratio of 97:3 (see SI, Fig. S5) by the conjugate addition. Reduction and elongation led to compound **3**, into which the second stereogenic center was introduced similarly to form compound **4** with an *R*/*S* ratio of 93:7 (calculated back from the final product). Reduction of the esters to alcohol **5** and conventional Grignard-elongation delivered compound **7** that, after deprotection and oxidation, gave (2*S*,4*S*)-acid **9** as the major product, which after esterification with 1-tetradecanol furnished ester **10** (Fig. 6).

Usually, multiple syntheses of different stereoisomers are required for elucidation of the absolute configuration of compounds with multiple stereogenic centers (see e. g. Schulz et al. 2004). We tried to solve this problem here with only one stereoselective synthesis, taking advantage of the controlled stepwise introduction of the stereogenic centers as explained in the next section.

### Stereochemistry of Wax Esters

**Fig. 7:**
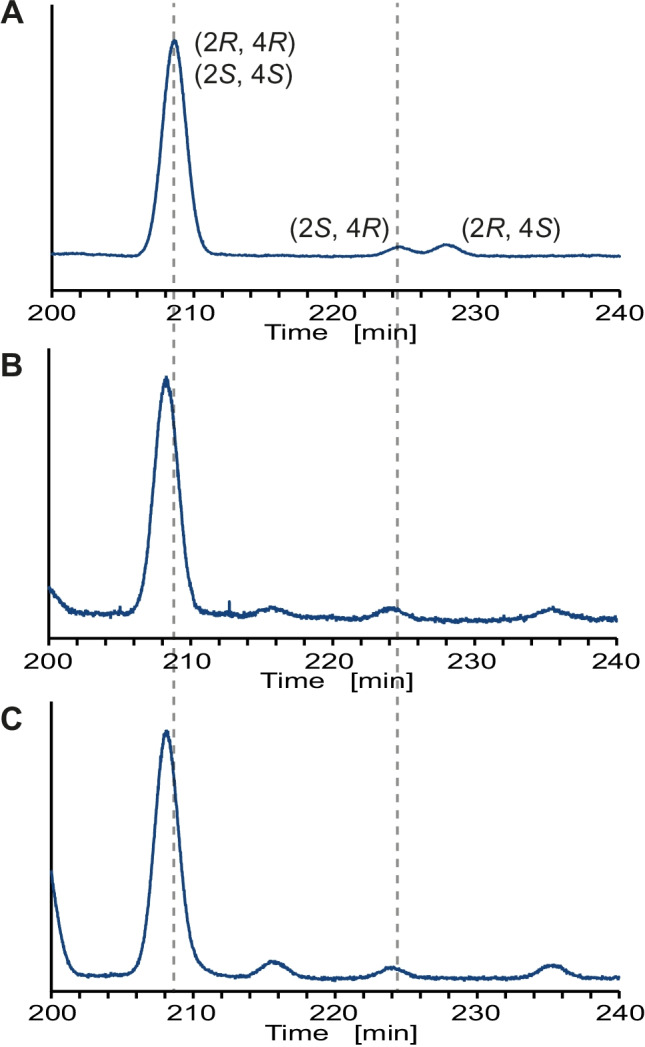
Resolution of the methyl ester of 2,4-dimethylheptadecanoic acid (**9**) on a chiral GC phase. Separation was performed using a Hydrodex β-6TBDM phase (30.0 m × 0.25 mm, 1.5 mL/min H_2_, initial temp. 50 °C then 10 °C min^−1^ to 125 °C holding time for 240 min, then with 10 °C min^−1^ to final temp. 230 °C). **A**: methyl ester of synthetic (2*S*,4*S*)-2,4-dimethylheptadecanoic acid (**9)**. **B**: methyl ester of the transesterified sample of a body extract of male *Argiope bruenichi*. **C**: same with female *A. bruenichi*

GC showed two separated peaks in the final product **10** in a diastereomeric ratio of 91:9 (Fig. S3). The major, first eluting peak was the *syn*-diastereomer (2*S*,4*S*/2*R*,4*R*), while the smaller, second one was the *anti*-diasteromer (2*S*,4*R*/2*R*,4*S*). The methyl ester of **9** showed similar separation. The stereoisomeric mixture of the methyl esters of **9** was then separated on a chiral Hydrodex β-6TBDM phase (Fig. 7). Peak assignment was performed using the enantiomeric excess (ee) data from the stereoselective addition. Therefore, the largest peak was the (2*S*,4*S*)-enantiomer (0.93 × 0.97 = 0.9021 relative peak area, ee 99.8%), while the smallest had a (2*R*,4*R*)-configuration (0.07 × 0.03 = 0.0021), likely not detectable due to its low abundance. The (2*R*,4*S*)-enantiomer (0.07 × 0.97 = 0.0679, ee 41.8%) was slightly more concentrated than the (2*S*,4*R*)-enantiomer (0.93 × 0.03 = 0.0279). The ee data nicely showed that the consecutive introduction of chiral centers leads to a *syn*-product of very high ee due to the inherent ee amplification, while it is the opposite for the *anti*-diastereomer. The *anti*-enantiomers are well separated, while resolution of the *syn*-enantiomers was not possible (see Fig. S7). Comparison with the transesterified natural extracts revealed the natural *anti*-diastereomer to have the (2*S*,4*R*)-configuration. This indicates that the natural *syn*-diastereomer can be assigned the (2*R*,4*R*)-stereochemistry because the (4*R*)-stereochemistry is fixed during biosynthesis, in contrast to the configuration at C-2. For a detailed discussion see the Supporting Information. The formation of the *anti*-diastereomer might be explained by partial epimerization of C-2 during biosynthesis of the esters. Epimerization during transesterification with TMSH is unlikely because of the reaction mechanism and the occurrence of diastereomers of some esters even in the original samples (Table 1).

In summary, over 180 cuticular compounds were detected and most of them were identified using the methods described above. While the number of hydrocarbons (72) and wax esters (75) was almost equal, the esters dominated in cuticular extracts of both females (ratio amounts esters/hydrocarbons 64:28) and males (68:13), while both compound classes occurred in equal amounts in webs of females (44:41).

## Discussion

In this study, we identified for the first time wax esters with a bishomomethyl-branched acid head group that constitute the major portion of lipids on the cuticle of both sexes and web silk of females of *A. bruennichi*. These cuticular compounds likely play a role in kin recognition, because family differences were observed within the wax esters, while cuticular hydrocarbons showed lower variation (Weiss and Schneider 2021). We developed an analytical procedure that can reliably be used to analyze and characterize these esters, based on previous work (Chinta et al. 2016). Furthermore, we established a synthetic procedure that allows access to the enantiomers of each ester and enables determination of the absolute configuration of the esters.

Wax esters have previously been described as cuticular compounds of spiders and scorpions (Trabalon and Bagnères 2010). Propyl esters of long-chain multiply methyl-branched acids occur in complex mixtures on *Anelosimus eximus* (Bagnères et al. 1997), while a few shorter, sex-specific esters such as 2,8-dimethylundecyl 2,8-dimethylundecanoate or 14-methylheptadecyl 4-methylheptanoate dominate the cuticular wax of *Argyrodes elevatus* (Chinta et al. 2016). In contrast, the bishomomethyl-branched esters reported here represent a unique group of compounds that have not been reported from other arthropods. Nevertheless, they do not seem to be specific to *Argiope*, because we have also detected such compounds in the spider *Pholcus phalangoides* (S. Schulz, unpublished).

The structures of the hydrocarbons identified here are not very different from those found in other spiders. An enhanced concentration of 2-methyl-branched alkanes with an even number of carbons was observed, in line with reports from other spider species (Schulz 2001, 2013), a trait usually not observed within insect hydrocarbons. A close similarity in compound structures between samples from females and males is evident, but sex differences exist in the number of compounds, which is lower in males, and the relative proportions of the components. Consequently, cuticular profiles of males and females were clearly separated by discriminant analyses based on relative peak areas, while the profile of silk from females more closely resembled cuticular extracts from females. Specifically, females showed a larger number of trimethyl-substituted esters and a slightly higher molecular weight, ranging from 480 to 564 amu, while those in males ranged from 466 to 536 amu. Both sexes share tridecyl *syn*-2,4-dimethylnonadecanoate (522 amu) as a major component. However, females also have considerable amounts of tridecyl *syn*-2,4-dimethylicosanoate and tridecyl *syn*-2,4-dimethylhenicosanoate (536 amu), whereas males possess more tridecyl 2,4-dimethyloctadecanoate (494 amu) and tridecyl *syn*-2,4-dimethylheptadecanoate (508 amu). Hence females are characterized by larger esters than males, but we also found male specific wax esters, namely tridecyl and tetradecyl esters of octadecanoic and several 2,4,14-trimethylalkanoic acids.

In summary, we have clarified the complex cuticular chemistry of *A. bruennichi*. With these data and synthetic material, behavioral assays can be planned to elucidate which signals are used for kin recognition. Although synthesis of a complete array of esters seems out of reach, addition of individual esters to manipulate cuticular chemistry might be a promising approach for further research.

## Supplementary Information

Below is the link to the electronic supplementary material.

Supplementary file1 (PDF 669 KB)

## Data Availability

Not applicable.
