# Supplementary material for: Identification of Cuticular and Web Lipids of the Spider Argiope bruennichi
Source: J Chem Ecol. 2022 Jan 10;48(3):244–62. doi: 10.1007/s10886-021-01338-y (PMC8934766; doi:10.1007/s10886-021-01338-y)
Supplement: Supplementary file 1 — Supplementary file1 (PDF 669 KB) [file 10886_2021_1338_MOESM1_ESM.pdf]

IDENTIFICATION OF CUTICULAR AND WEB LIPIDS OF THE SPIDER *Argiope bruennichi*

MORITZ GERBAULET,[1] ANTON MÖLLERKE,[1] KATHARINA WEISS,[2] SATYA CHINTA,[1,3]
JUTTA M. SCHNEIDER,[2] STEFAN SCHULZ[1,*]

[1] *Institute of Organic Chemistry, TU Braunschweig, Hagenring 30, 38106 Braunschweig, Germany*

[2] *Institute of Zoology, University of Hamburg, Martin-Luther-King Platz 3, 20146 Hamburg, Germany*

[3] *Current address: U.S. Department of Agriculture, Agricultural Research Service, 1600-1700 SW 23rd Drive, Gainesville, FL 32608, USA*

Mass spectra of synthetic material

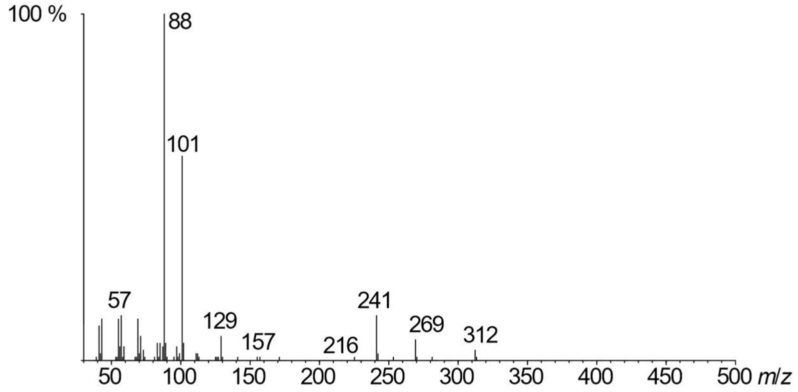

Fig. S1 Mass spectrum of synthetic methyl 2,4-dimethylheptadecanoate.

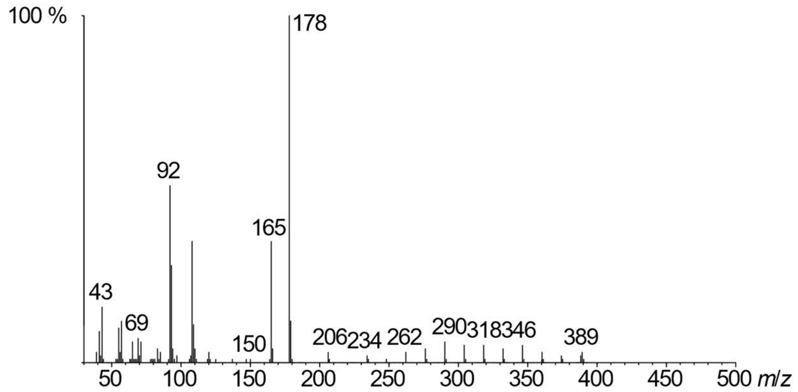

Fig. S2 Mass spectrum of synthetic 3-pyridinylmethyl 2,4-dimethylheptadecanoate.

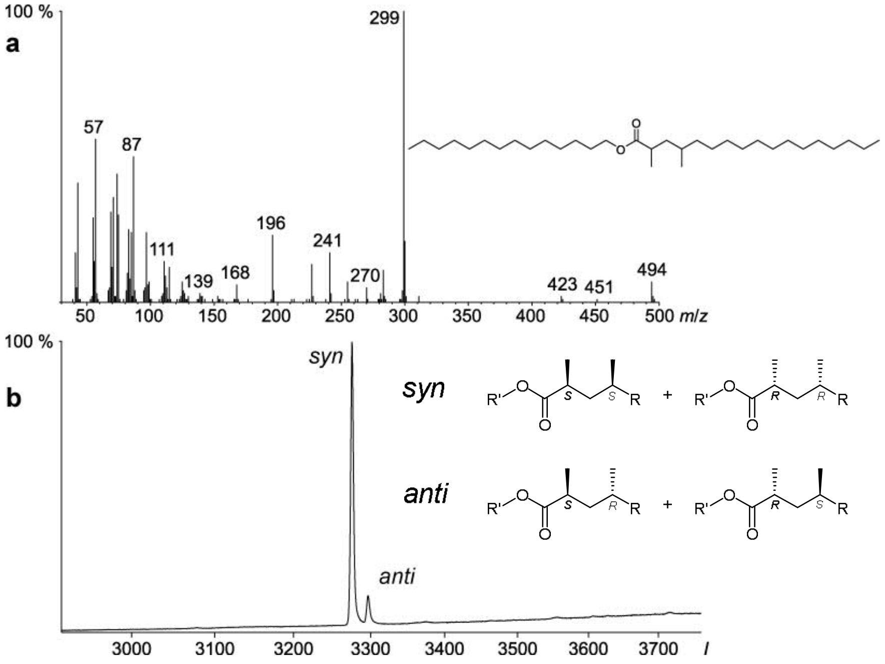

Fig. S3 Mass spectrum of synthetic **10** (**a**) and TIC of the diastereomeric mixture on a HP5-MS phase.

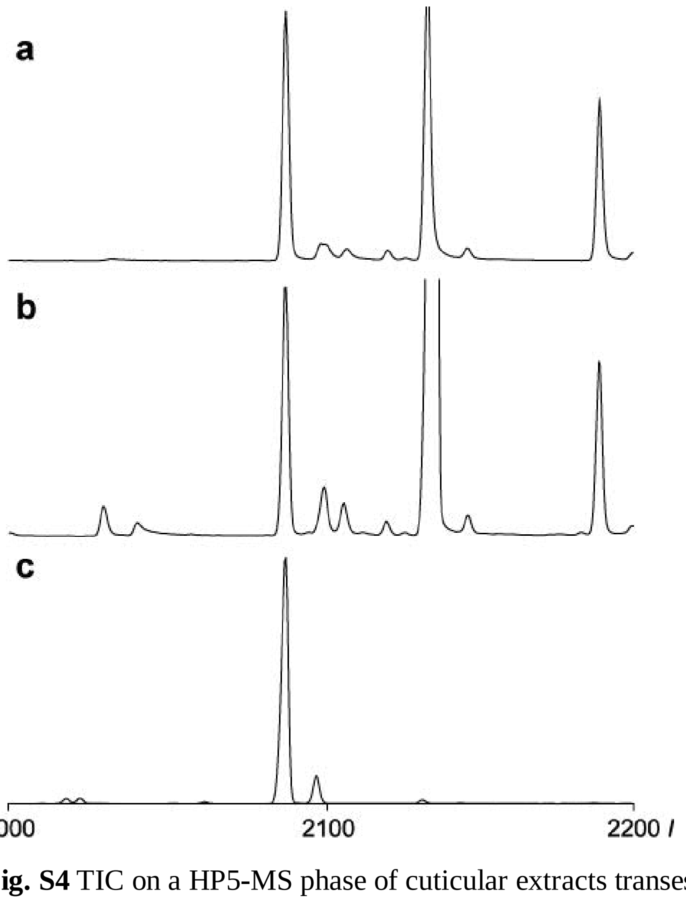

Fig. S4 TIC on a HP5-MS phase of cuticular extracts transesterified into methyl esters. a: male; b: female; c: synthetic methyl *syn* and *anti* methyl 2,4-dimethylheptadecanoate.

Determination of the absolute configuration

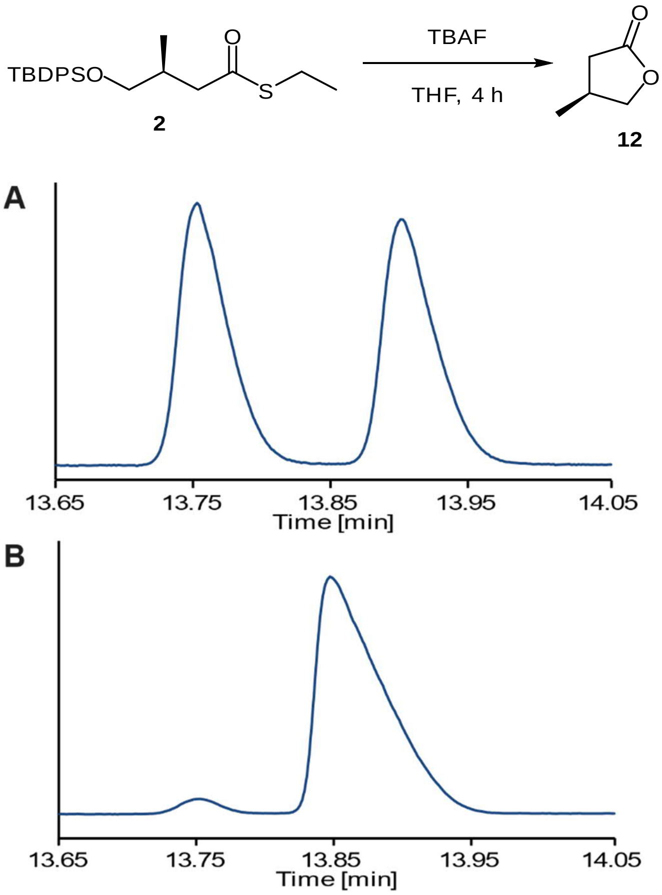

Fig. S5 For the determination of the enantiomeric purity of building block **2** this compound was transformed into lactone **12** with tetrabutylammonium fluoride. Separation was performed by GC on a chiral phase [BetaDex^{TM} 225 (30.0 m x 0.25 mm, initial temp. 50 °C then 10 °C min^{-1} to final temp. 160 °C)]. Retention time, 13.75 min (minor), 13.85 (major) showed an ee of 94% (Lit.: 98% ee Horst et al. 2007). **A**: racemic lactone **12**. **B**: synthesized lactone **12**.

Why do the major natural esters likely show a (2*R*,4*R*)-configuration?

The biosynthesis of fatty acids has been well studied and is fundamentally identical in all animals (Schomburg and Michal 2012). The biosynthesis of acid **9** starts with propionyl-CoA (Fig. S5) followed by condensation of malonate units via the multi-enzyme complex of the fatty acid synthase to arrive at the acyl-carrier-protein bound tridecanoate **A** (Morgan 2010). The addition of methylmalonate leads to the unsaturated species **B** along the typical condensation-reduction-elimination elongation pathway. The acyl carrier protein, serving as a robotic arm of the enzyme complex, now presents this to the hydrogenation domain leading to (*R*)-2-methylpentadecanoate (**C**) defining the C-4 stereogenic center in **9**. This enzyme must be highly stereoselective, because we do not see any signal of the (2*R*,4*S*)-stereoisomer of the methyl ester of **9** (Fig. 6bc). In the next elongation round the same hydrogenation domain leads to (2*R*,4*R*)-2,4-dimethylpentadecanoate **E**, with identical stereoselectivity as before. If the other epimer would be formed, an *anti*-configuration (2*R*,4*S* or 2*S*,4*R*) of the two methyl groups would result, but GC showed that the *cis*-diastereomer (2*R*,4*R* or 2*S*,4*S*) is the major natural compound (Fig.

6). Therefore, we can conclude that the major ester has a (2*R*,4*R*)-configuration. In further processing the thioesterase releases the fatty acid, which has to be activated to an acyl-coenzyme A (CoA) ester by a ligase to form the wax esters (Schomburg and Michal 2012), likely by a specific ester synthase. During all these processes C-2 of the acid is chemically activated due to the neighboring carbonyl group, in contrast to C-4. Thus, a small part of the material might be epimerized, thus leading to the small amount of the (2*S*,4*R*)-compound detected in the samples (Fig. S 4). Chiral GC of racemic methyl ester of **9** (Fig. S6) showed that the *syn*-stereoisomers cannot be separated, because the natural (2*R*,4*R*)-isomer and the synthetic (2*S*,4*S*)-isomer have the same retention time, in contrast to the *anti*-stereoisomers.

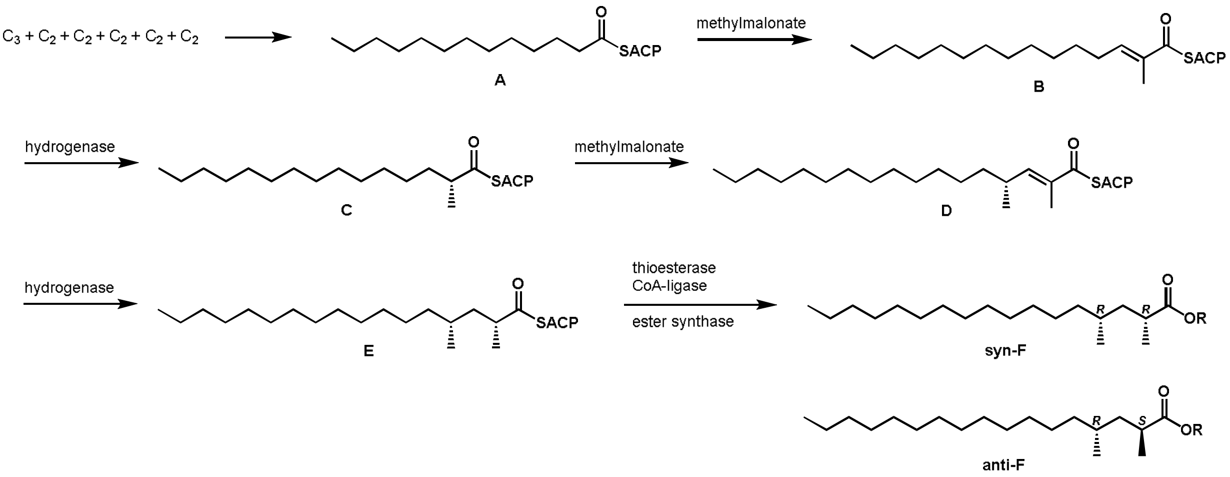

Fig. S6 Biosynthetic pathway to the formation of wax esters in *A. bruennichi.*

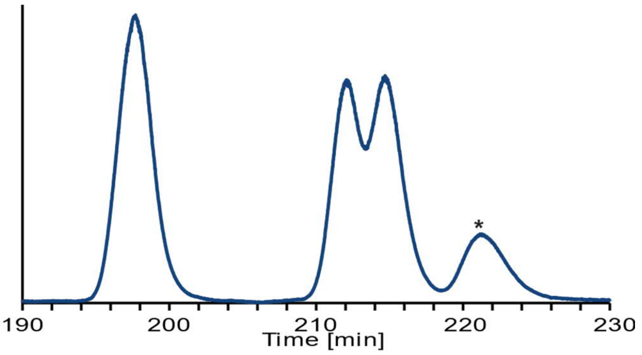

Fig. S7 Separation of the racemic methyl ester of 2,4-dimethylheptadecanoic acid (**9)** on a chiral Hydrodex β-6TBDM phase (30.0 m x 0.25 mm, 1.5 mL/min H_2, initial temp. 50 °C then 10 °C min^{-1} to 125 °C holding time for 240 min, then with 10 °C min^{-1} to final temp. 230 °C). The peak marked with an * is an impurity. Peak identity was confirmed by MS.

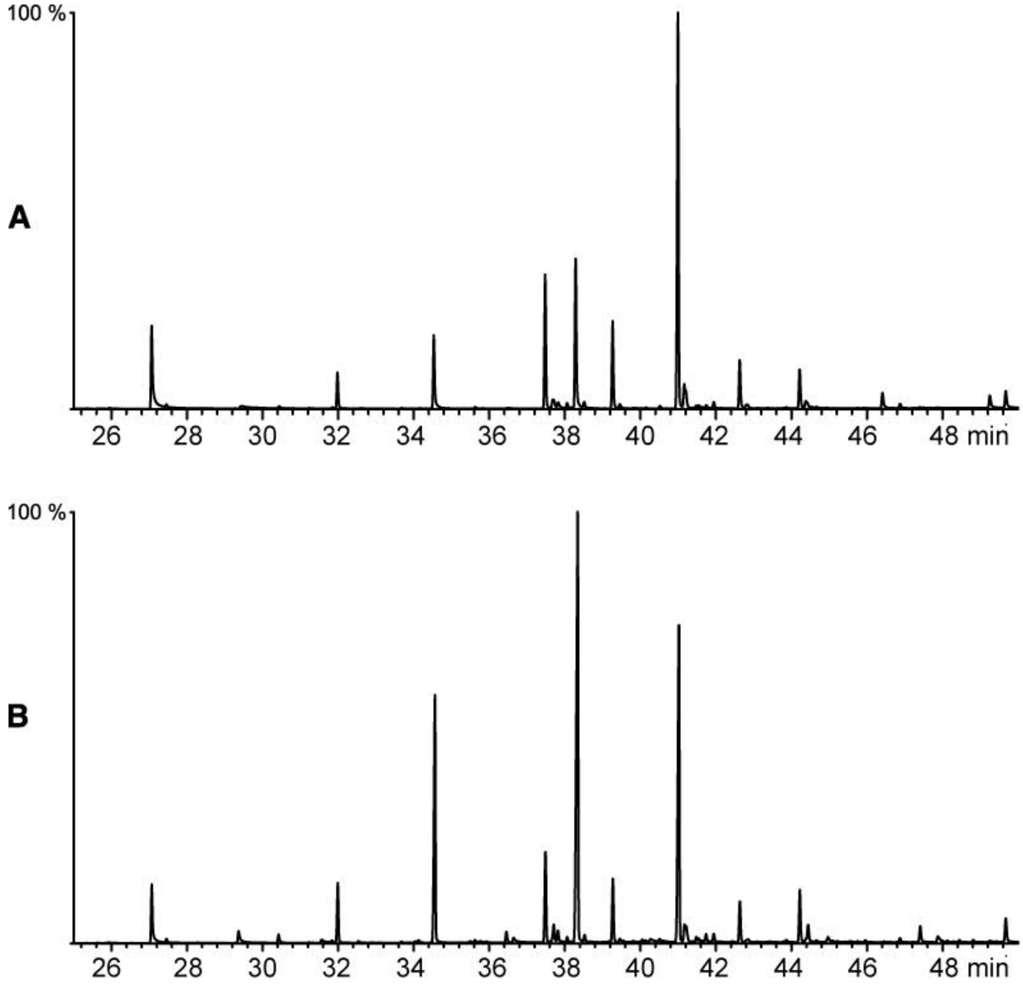

Fig. S8 TIC of methyl ester derivatized extract of male (A) and female (B) silk of *A. bruennichi*.

Table S1: Methyl esters and alcohols obtained after transesterification of male silk extracts.

I	RT [min]	compound
1576	27.0675	1-tridecanol
1680	29.4236	1-tetradecanol
1727	30.4380	methyl tetradecanoate
1800	31.9845	octadecane
1884	33.6907	methyl 2,4-dimethylpentadecanoate
1927	34.5356	methyl hexadecanoate
1984	35.6311	methyl 2,4-dimethylhexadecanoate
2083	37.4836	*syn*-methyl 2,4-dimethylheptadecanoate
2094	37.6817	*anti*-methyl 2,4-dimethylheptadecanoate
2128	38.2910	methyl octadecanoate
2141	38.5221	methyl 2,4,14-trimethylheptadecanoate
2152	38.7172	methyl 2,4,16-trimethylheptadecanoate
2184	39.2702	*syn*-methyl 2,4-dimethyloctadecanoate
2194	39.4623	*anti*-methyl 2,4-dimethyloctadecanoate
2235	40.1518	methyl 2,4,14-trimethyloctadecanoate
2257	40.5195	methyl 2,4,16-trimethyloctadecanoate
2285	40.9982	*syn*-methyl 2,4-dimethylnonadecanoate
2295	41.1662	*anti*-methyl 2,4-dimethylnonadecanoate
2314	41.4844	methyl 2,4,10-trimethylnonadecanoate
2318	41.5557	methyl 2,4,8-trimethylnonadecanoate
2343	41.9473	methyl 2,4,14-trimethylnonadecanoate
2385	42.6339	*syn*-methyl 2,4-dimethyleicosanoate
2397	42.8267	*anti*-methyl 2,4-dimethyleicosanoate
2459	43.8007	methyl 2,4,14-trimethyleicosanoate
2485	44.2193	*syn*-methyl 2,4-dimethylheneicosanoate
2496	44.3844	*anti*-methyl 2,4-dimethylheneicosanoate

Table S2: Methyl esters and alcohols obtained after transesterification of female silk extracts.

RI	RT [min]	compound
1576	27.0690	1-tridecanol
1677	29.3650	1-tetradecanol
1726	30.4230	methyl tetradecanoate
1780	31.5635	1-pentadecanol
1800	31.9919	octadecane
1827	32.5374	methyl pentadecanoate
1883	33.6742	methyl 2,4-dimethylpentadecanoate
1928	34.5626	methyl hexadecanoate
1983	35.6213	methyl 2,4-dimethylhexadecanoate
2027	36.4534	methyl heptadecanoate
2083	37.4888	*syn*-methyl 2,4-dimethylheptadecanoate
2094	37.6794	*anti*-methyl 2,4-dimethylheptadecanoate
2115	38.0613	methyl 2,4,8-trimethylheptadecanoate
2130	38.3149	methyl octadecanoate
2142	38.5273	methyl 2,4,14-trimethylheptadecanoate
2152	38.7126	methyl 2,4,16-trimethylheptadecanoate
2184	39.2731	*syn*-methyl 2,4-dimethyloctadecanoate
2194	39.4584	*anti*-methyl 2,4-dimethyloctadecanoate
2225	39.9837	methyl 2,4,6-trimethyloctadecanoate
2234	40.1427	methyl 2,4,14-trimethyloctadecanoate
2256	40.5127	methyl 2,4,16-trimethyloctadecanoate
2286	41.0214	*syn*-methyl 2,4-dimethylnonadecanoate
2295	41.1707	*anti*-methyl 2,4-dimethylnonadecanoate
2314	41.4836	methyl 2,4,10-trimethylnonadecanoate
2318	41.5496	methyl 2,4,8-trimethylnonadecanoate
2330	41.7395	methyl 2,4,6-trimethylnonadecanoate
2342	41.9435	methyl 2,4,14-trimethylnonadecanoate
2385	42.6339	*syn*-methyl 2,4-dimethyleicosanoate
2397	42.8470	*anti*-methyl 2,4-dimethyleicosanoate
2458	43.7901	methyl 2,4,16-trimethylnonadecanoate
2485	44.2216	*syn*-methyl 2,4-dimethylheneicosanoate
2496	44.3844	*anti*-methyl 2,4-dimethylheneicosanoate
2531	44.9186	methyl 2,4,6-trimethyleicosanoate

Synthetic procedures

General Experimental Procedures. All reactions were performed in oven-dried glassware under a nitrogen atmosphere. Solvents were dried according to standard procedures. Column chromatography: silica 60 (0.063–0.200 mm, 70–230 mesh ASTM). Thin layer chromatography (TLC): Polygram® SIL G/UV silica 60, 0.20 mm. Compounds were stained with potassium permanganate solution. NMR spectra were recorded either on Avance III HD 300N (^{1}H-NMR: 300 MHz, ^{13}C-NMR: 76 MHz), DRX 400 (^{1}H-NMR: 400 MHz, ^{13}C-NMR: 101 MHz), AVII 400 (^{1}H-NMR: 400 MHz, ^{13}C-NMR: 101 MHz) or AVII 600 (^{1}H-NMR: 600 MHz) instruments. Data are reported as follows: chemical shifts, multiplicity (s = singlet, d = doublet, t = triplet, q = quartet, m = multiplet), coupling constants (Hz). IR spectra were measured on a Bruker Tensor 27 (diamond-ATR). Mass spectra were recorded with a combination of an Agilent Technologies 5977B gas chromatograph connected to an Agilent Technologies 8860 Series MSD. The enantiomeric excess of chiral compounds was determined by GC on BetaDex™ 225 or Hydrodex β-6TBDM [(30.0 m x 0.25 mm) phases, operated with H_2 as carrier gas with a flow of 1.5 ml min. Optical rotation was determined with the help of an MCP 150 polarimeter (Anton Paar) with a cell length of 1 cm (*c* given in mg/mL).

Preparation of (*S*-ethyl 2-(triphenyl-λ^{5}-phosphaneylidene)ethanethioate) (15)

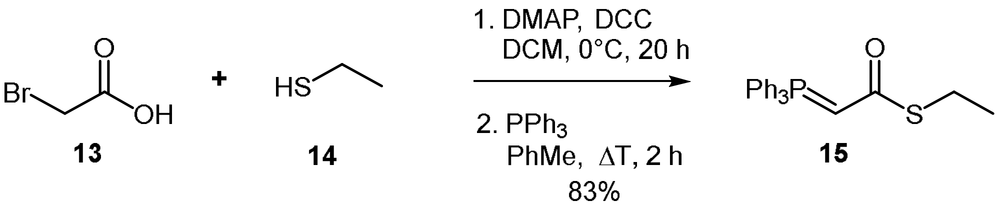

The synthesis is based on a protocol by Thielman *et al.* (Thielman et al. 2020). Ethanethiol (**14**, 140.34 mmol, 8.72 g, 1.3 eq.) and *N*,*N*-dimethylaminopyridine (DMAP, 10.80 mmol, 1.32 g, 0.1 eq.) were added to a solution of bromoacetic acid (**13**, 107.95 mmol, 15.00 g, 1.0 eq.) in CH_2Cl_2 (250 mL) at 0 °C. After stirring 15 min dicyclohexylcarbodiimide (DCC, 113.35 mmol, 23.39 g, 1.1 eq.) was added at 0 °C over a period of 3 h. The reaction mixture was stirred 1.5 h at 0 °C, was allowed to warm to room temperature and stirred for additional 20 h. Afterwards, the mixture was filtered through celite and washed with CH_2Cl_2. The solvent was separated under reduced pressure. The residue was dissolved in benzene (150 mL) triphenylphosphine (PPh_3, 113.35 mmol, 29.73 g, 1.1 eq.) was added. The mixture was heated to reflux for 2 h under a nitrogen atmosphere and stirred for 20 h at room temperature. The formed precipitate was separated through filtration and suspended in CH_2Cl_2 (150 mL). Sat. Na_2CO_3 solution (150 mL) was added to the mixture. After stirring for 40 min under a nitrogen atmosphere the phases were separated, the aqueous phase was extracted three times with CH_2Cl_2 (50 mL), the combined organic phases were dried over Na_2SO_4, and the solvent was separated under reduced pressure. The product was purified by precipitation from pentane/CH_2Cl_2 (2:1), followed by repeated concentration

and precipitation through addition of pentane from the mother liquor to afford **15** as slightly yellow crystals (32.71 g, 83%). FT-IR: v / cm^{-1} = 1579, 1565, 1481, 1436, 1408, 1337, 1318, 1261, 1184, 1103, 1085, 1029, 997, 973, 871, 800, 752, ,713, 691, 663, 617, 549. ^{1}H-NMR: (400 MHz, $CDCl_3$) δ / ppm = 7.68 – 7.52 (m, 1H), 7.50 – 7.42 (m, 1H), 3.66 (d, J = 22.7 Hz, 1H), 2.84 (q, J = 7.4 Hz, 1H), 1.25 (t, J = 7.4 Hz, 1H). ^{13}C-NMR, DEPT: (101 MHz, $CDCl_3$) δ / ppm = 180.5 (C=O), 133.0 (CH_{Ar}), 132.9 (CH_{Ar}), 132.2 (CH_{Ar}), 132.1 (CH_{Ar}), 128.9 (CH_{Ar}), 128.8 (CH_{Ar}), 127.4 (C_{Ar}), 126.2 (CH_{Ar}), 47.6 (CH_2), 46.2 (CH_2), 23.1 (CH_2), 16.3 (CH_3).

Preparation of (2-((*tert*-butyldiphenylsilyl)oxy)ethan-1-ol) (17)

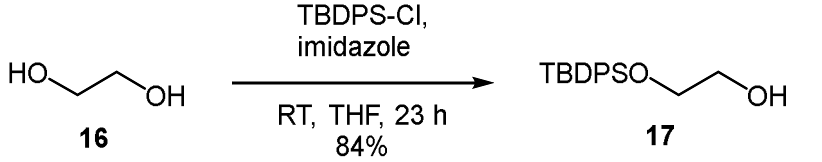

tert-Butyl(chloro)diphenylsilane was added to a solution of ethylene glycol (**16**) (231.39 mmol, 14.36 g, 6 eq.) and imidazole (42.42 mmol, 2.89 g, 1.1 eq.) in tetrahydrofuran (200 mL) over a period of 1 h under a nitrogen atmosphere. The reaction mixture was stirred at room temperature for 48 h. After quenching with H_2O (150 mL) the phases were separated and extracted two times with diethyl ether (200 mL). The combined organic phases were washed with H_2O, dried over Na_2SO_4. The solvent was removed under reduced pressure to afford **17** as a colorless oil (9.76 g, 84%). FT-IR: v / cm^{-1} = 3395, 3071, 3950, 2931, 2858, 1739, 1589, 1472, 1391, 1372, 1241, 1189, 1109, 1045, 1007, 999, 938, 880, 822, 737, 700, 688, 612, 557, 526. ^{1}H-NMR: (300 MHz, $CDCl_3$) δ / ppm = 7.80 – 7.68 (m, 1H), 7.51 – 7.37 (m, 1H), 3.85 – 3.77 (m, 1H), 3.77 – 3.67 (m, 1H), 2.32 (s, 1H), 1.13 (s, 2H). ^{13}C-NMR, DEPT: (76 MHz, $CDCl_3$) δ / ppm = 135.6 (CH_{Ar}), 133.4 (C_{Ar}), 129.9 (CH_{Ar}), 127.9 (CH_{Ar}), 65.1 (CH_2), 63.8 (CH_2), 27.0 (CH_3), 19.3 (C_q). EI-MS (70 eV): m/z (%) = 243 ([M-^{t}Bu]$^+$, 44), 199 ([M-^{t}Bu, -CH_2CH_2OH]$^+$, 93), 181 (22), 200 (22), 166 (19), 165 ([M-^{t}Bu-Ph]$^+$, 100), 139 (13), 123 (12), 105 (14), 77 (17).

Preparation of (*S*-ethyl (*E*)-4-((*tert*-butyldiphenylsilyl)oxy)but-2-enethioate) (19)

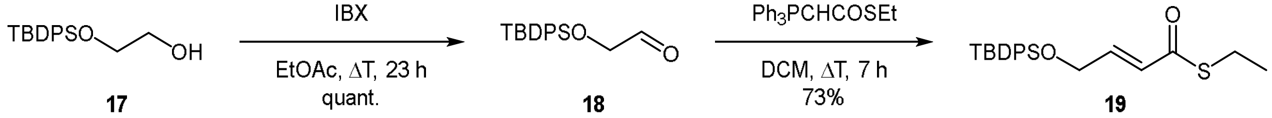

2-Iodoxybenzoic acid (IBX, 15.14 mmol, 4.24 g, 1.3 eq.) was added to a solution of 2-((*tert*-butyldiphenylsilyl)oxy)ethan-1-ol (**17**, 11.65 mmol, 3.50 g, 1.0 eq.) in ethyl acetate (100 mL). After heating to reflux for 23 h the mixture was cooled to room temperature and filtered through silica. The

solvent was removed under reduced pressure to afford **18** as a yellow oil (3.80 g, quant.). The crude aldehyde **18** was used in the next step without further purification. *S*-Ethyl 2-(triphenyl-λ^5-phosphaneylidene)ethanethioate (**15**, 30.77 mmol, 11.22 g, 1.3 eq.) was added to a solution of **18** (23.67 mmol, 7.07 g, 1.0 eq.) in CH_2Cl_2 (150 mL). The mixture was heated to reflux for 13 h undera nitrogen atmosphere and stirred at room temperature for additional 15 h. The solvent was removed under reduced pressure and the residue was purified by column chromatography ($AgNO_3$ treated silica, pentane/diethyl ether; 20:1) to afford **19** as a colorless oil (6.69 g, 73%). FT-IR: v / cm^{-1} = 3134, 3071, 2959, 22932, 2892, 2858, 1669, 1639, 1590, 1467, 1376, 1258, 1188, 1110, 1056, 1035, 1014, 948, 822, 782, 740, 700, 656, 617, 575. ^{1}H-NMR: (300 MHz, $CDCl_3$) δ / ppm = 7.65 (m, 4H), 7.48 – 7.35 (m, 6H), 6.89 (dt, J = 15.3, 3.4 Hz, 1H), 6.55 (dt, J = 15.3, 2.2 Hz, 1H), 4.35 (dd, J = 3.3, 2.3 Hz, 2H), 2.96 (q, J = 7.4 Hz, 2H), 1.29 (t, J = 7.4 Hz, 3H), 1.08 (s, J = 2.8 Hz, 9H). ^{13}C-NMR, DEPT: (76 MHz, $CDCl_3$) δ / ppm = 190.21 (C=O), 142.9 (CH), 135.6 (CH_{Ar}), 133.0 (C_{Ar}), 130.0 (CH_{Ar}), 128.0 (CH_{Ar}), 126.9 (CH), 62.9 (CH_2), 26.9 (CH_3), 23.3 (CH_2), 19.4 (C), 14.9 (CH_3). EI-MS (70 eV): m/z (%) = 328 (16), 243 (100), 199 (26) , 197 (15), 183 (32), 137 (20), 135 (25), 105 (18).

NMR Spectra

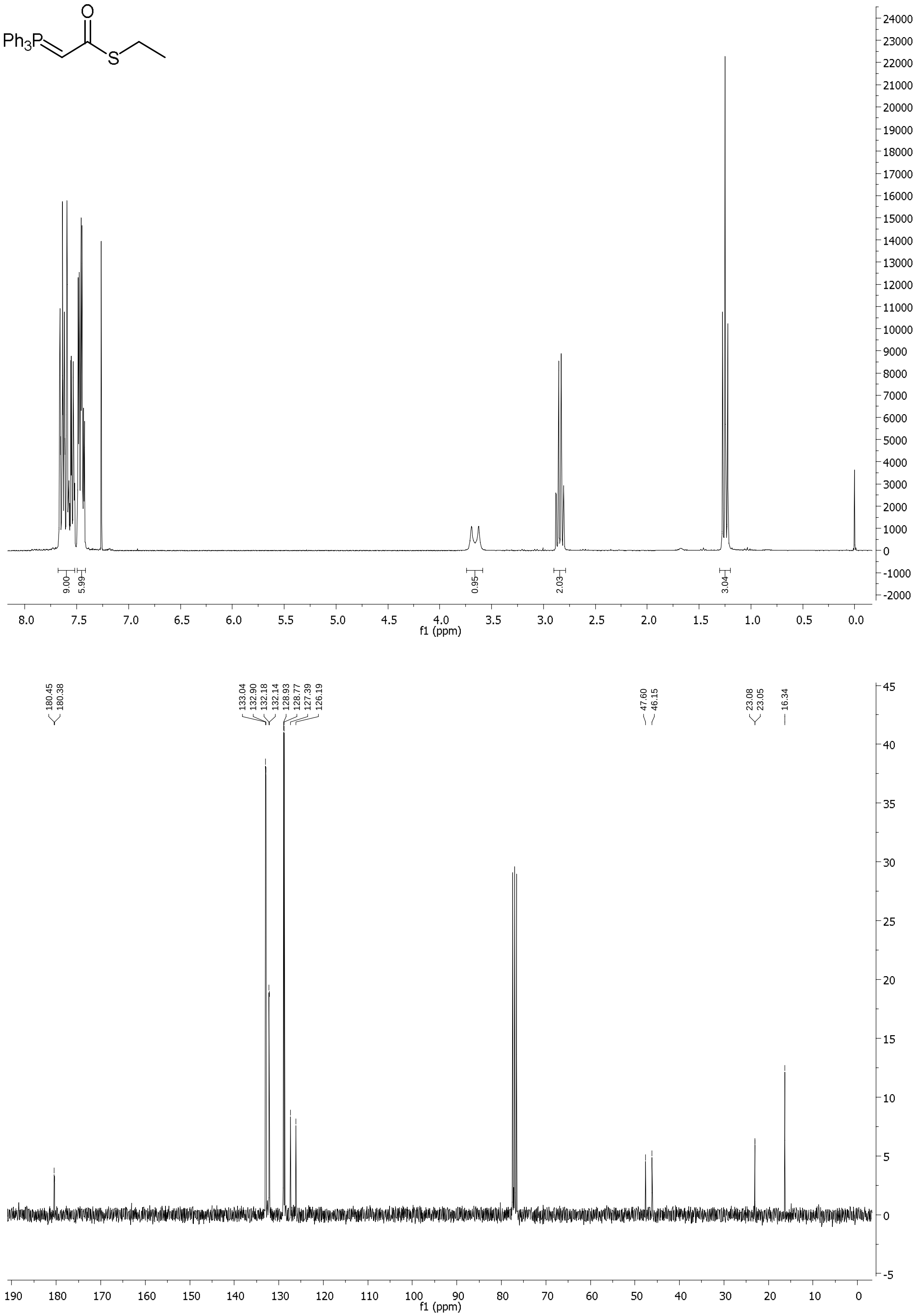

Fig. S9 ^{1}H-NMR (400 MHz, $CDCl_3$) and ^{13}C-NMR (101 MHz, $CDCl_3$) spectra *of S*-ethyl 2-(triphenyl-λ^5-phosphaneylidene)ethanethioate (**13**).

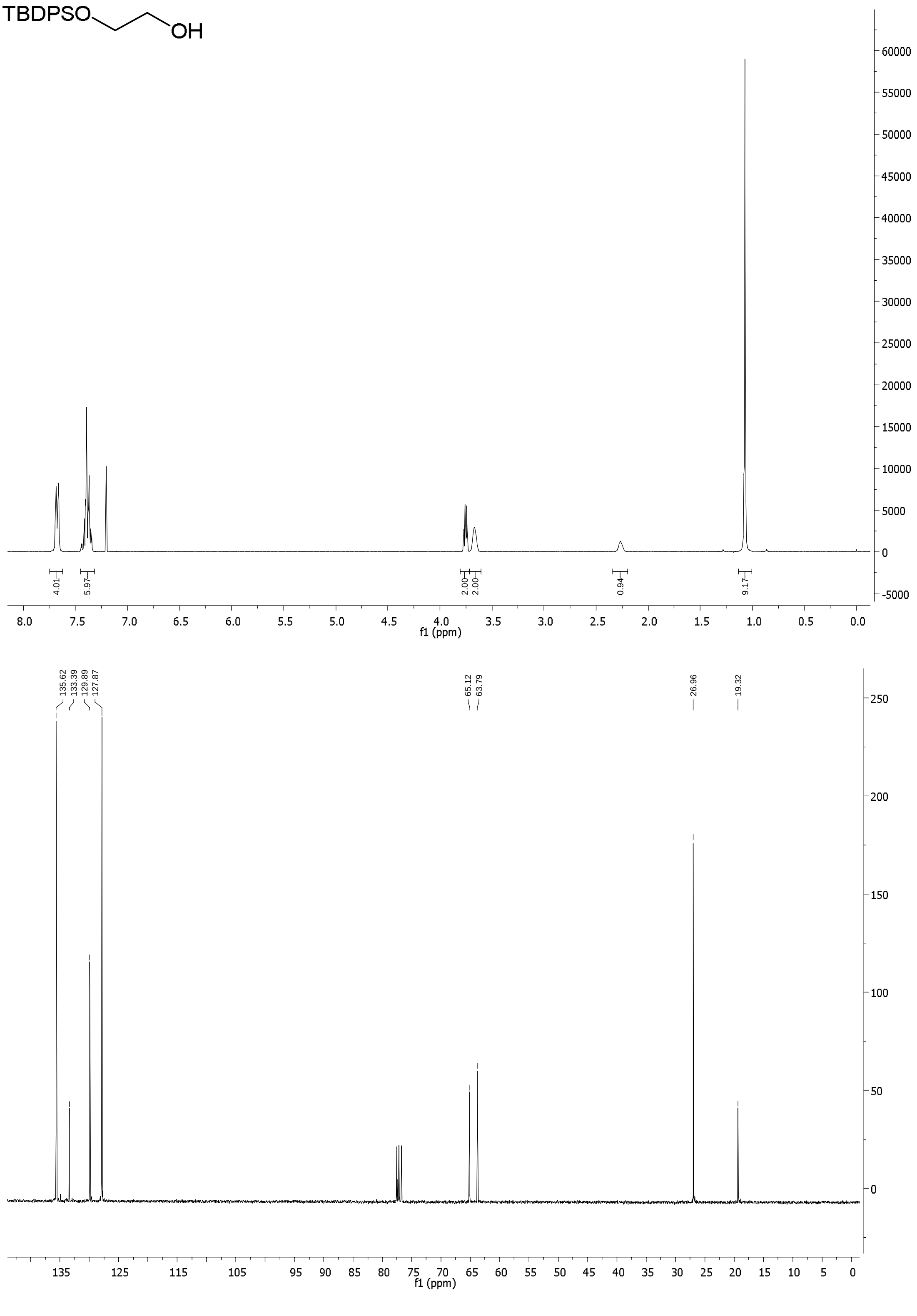

Fig. S10 ^{1}H-NMR (300 MHz, $CDCl_3$) and ^{13}C-NMR (76 MHz, $CDCl_3$) spectra of 2-((tert-butyldiphenylsilyl)oxy)ethan-1-ol (**15**).

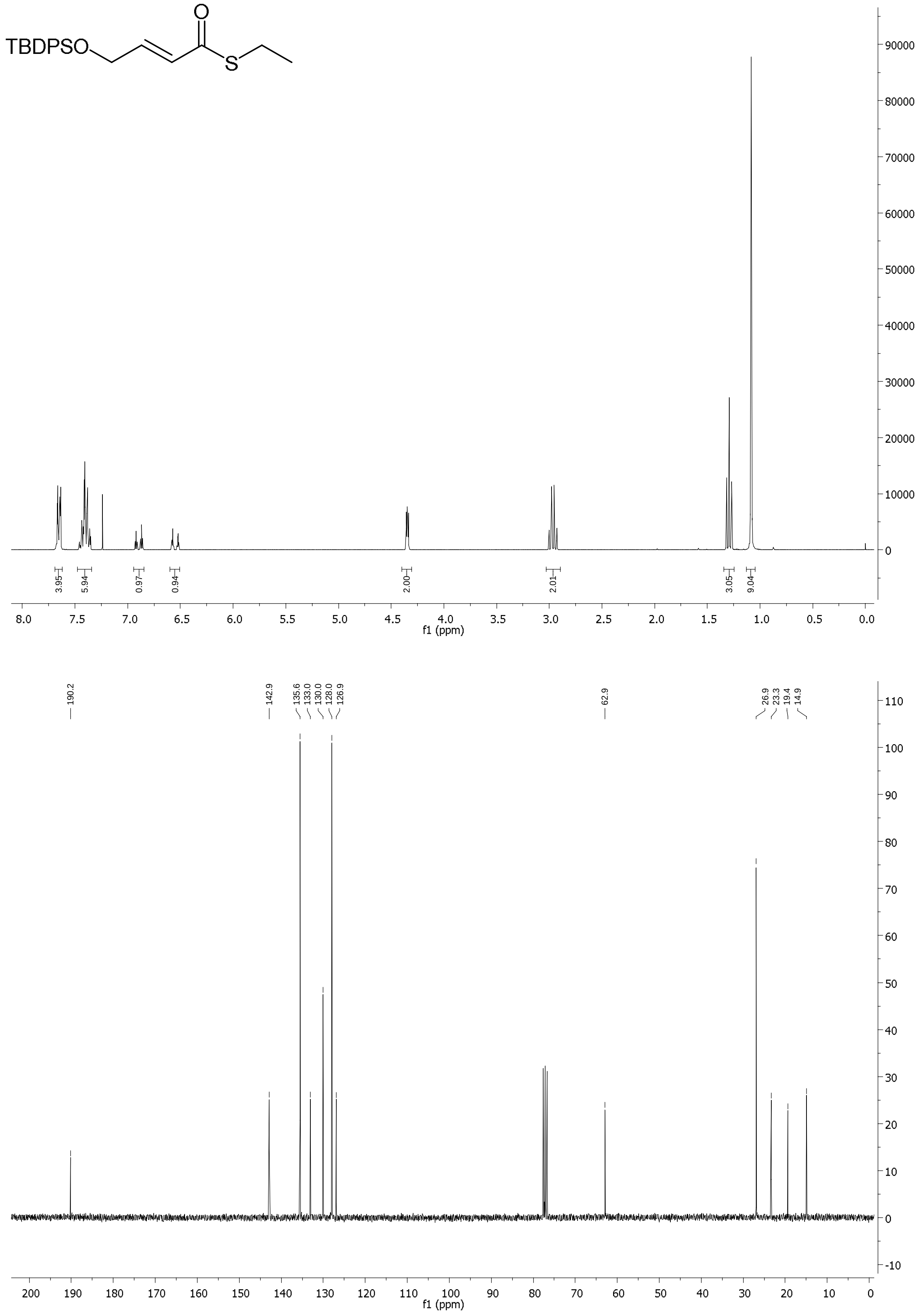

Fig. S11 ^{1}H-NMR (300 MHz, $CDCl_3$) and ^{13}C-NMR (76 MHz, $CDCl_3$) spectra of *S*-ethyl (*E*)-4-((*tert*-butyldiphenylsilyl)oxy)but-2-enethioate (**13**).

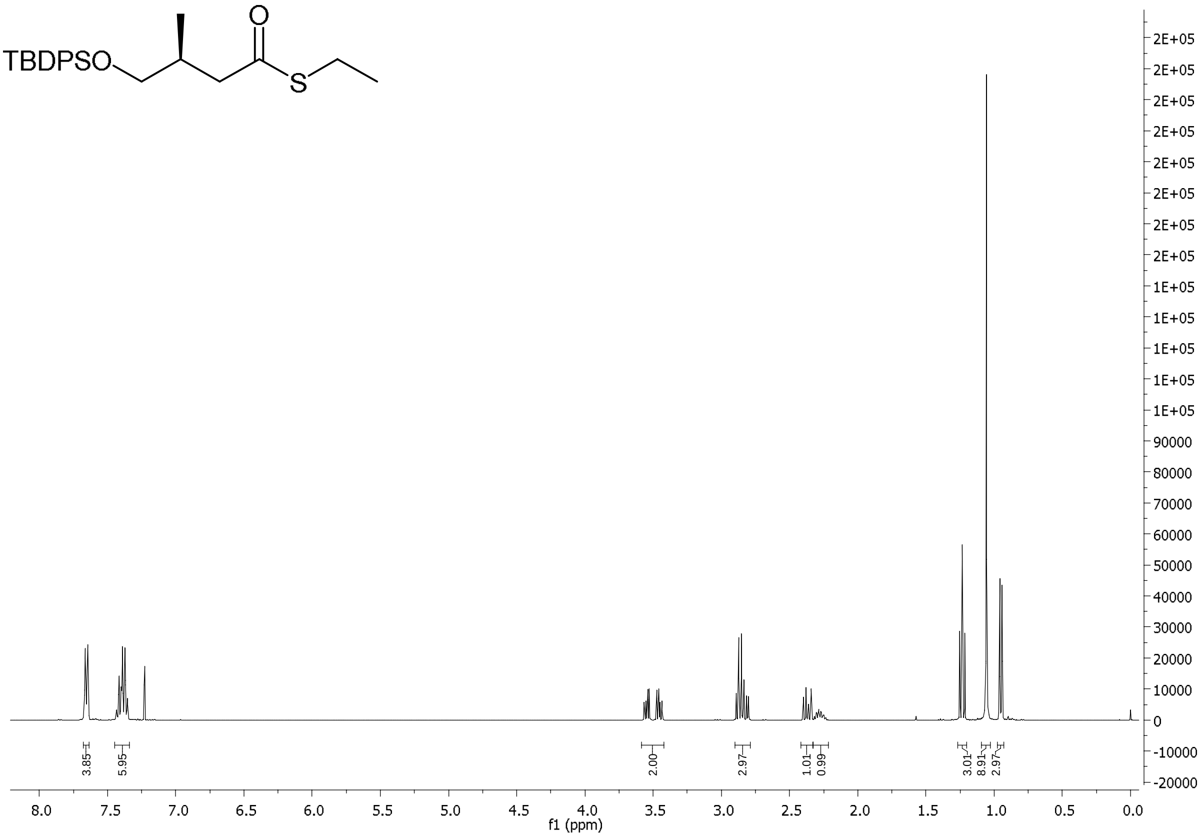

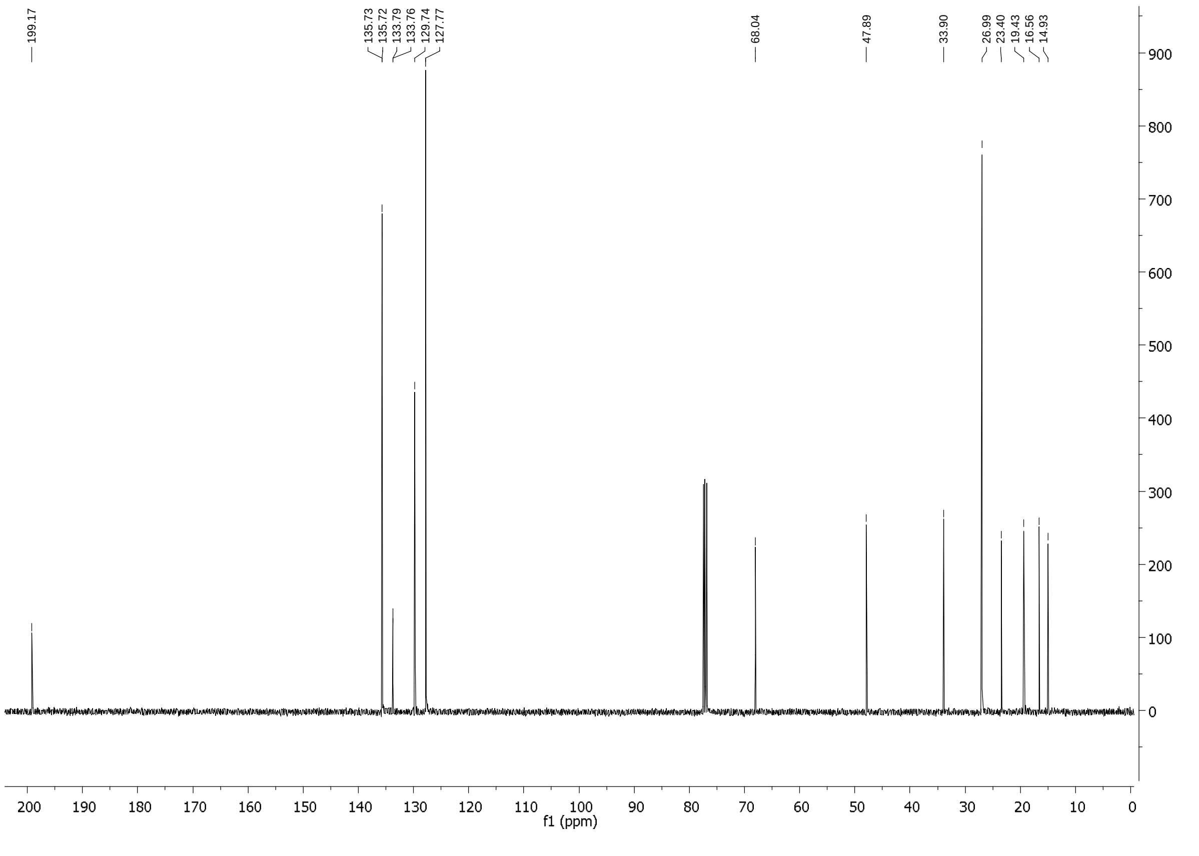

Fig. S12 ^{1}H-NMR (300 MHz, $CDCl_3$) and ^{13}C-NMR (76 MHz, $CDCl_3$) spectra of *S*-ethyl (*S*)-4-((*tert*-butyldiphenylsilyl)oxy)-3-methylbutanethioate (**2**).

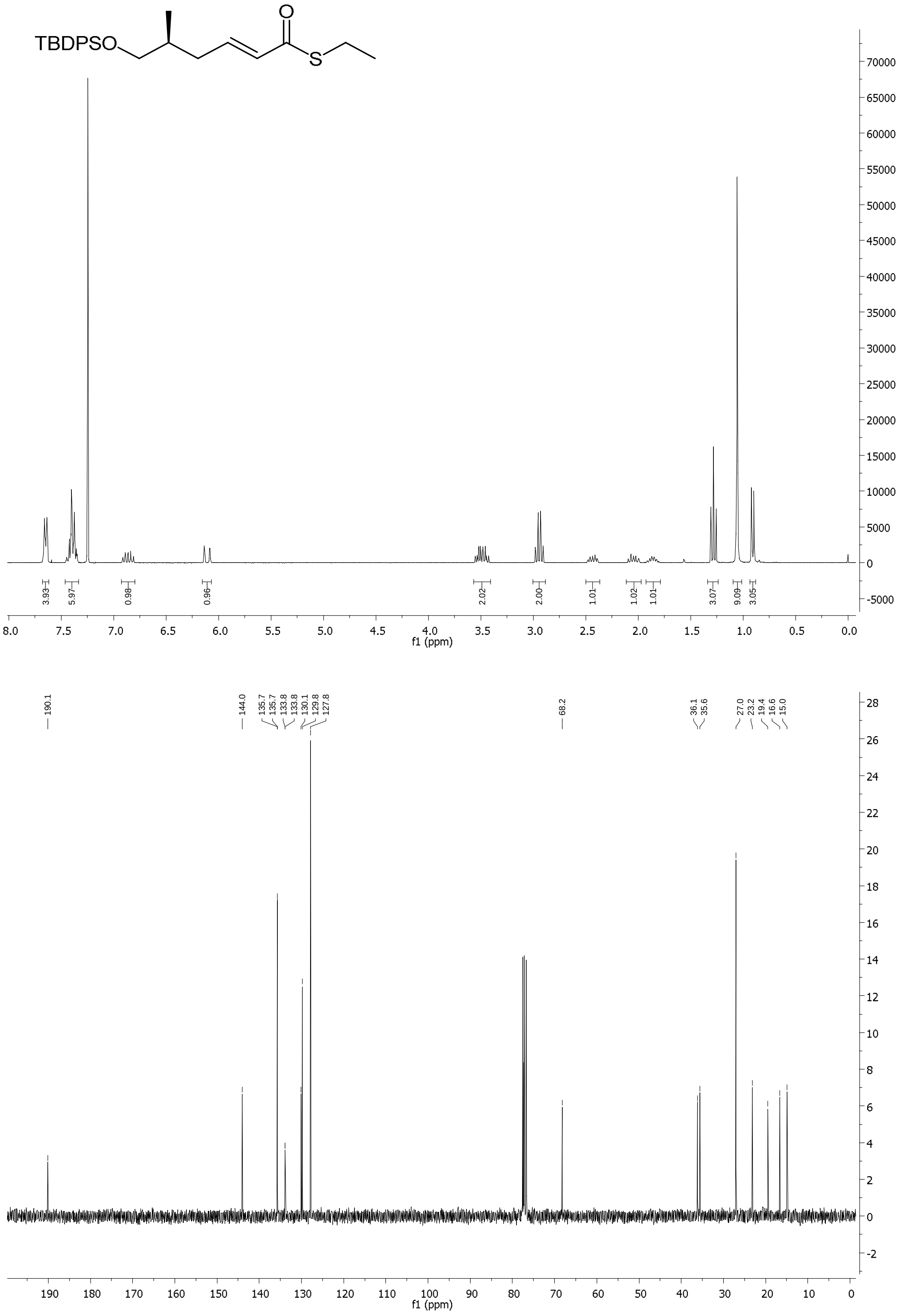

Fig. S13 ^{1}H-NMR (300 MHz, $CDCl_3$) and ^{13}C-NMR (76 MHz, $CDCl_3$) spectra of *S*-ethyl (*S*,*E*)-6-((*tert*-butyldiphenylsilyl)oxy)-5-methylhex-2-enethioate (**3**).

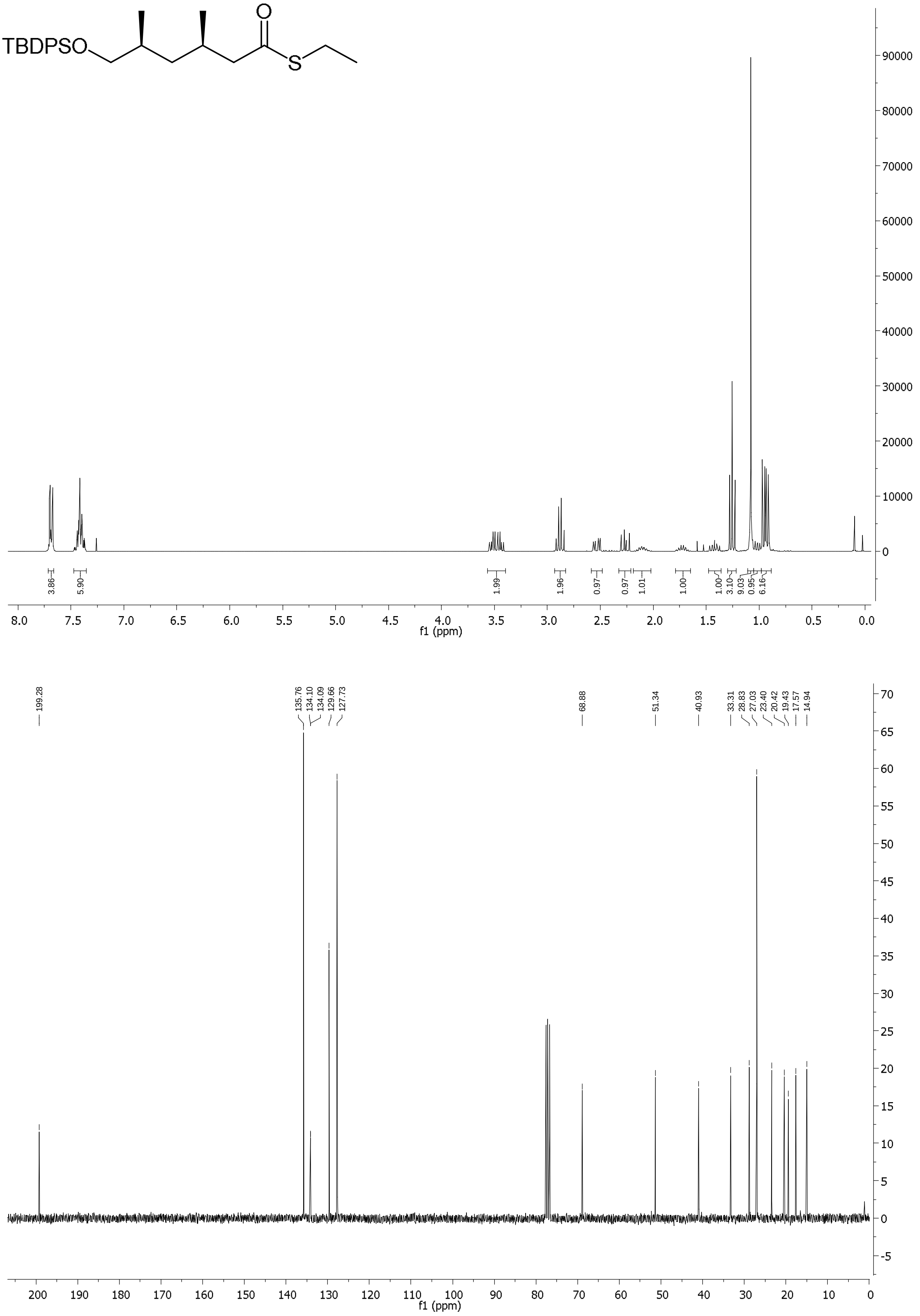

Fig. S14 ^{1}H-NMR (300 MHz, $CDCl_3$) and ^{13}C-NMR (76 MHz, $CDCl_3$) spectra of *S*-ethyl (3*R*,5*S*)-6-((*tert*-butyldiphenylsilyl)oxy)-3,5-dimethylhexanethioate (**4**).

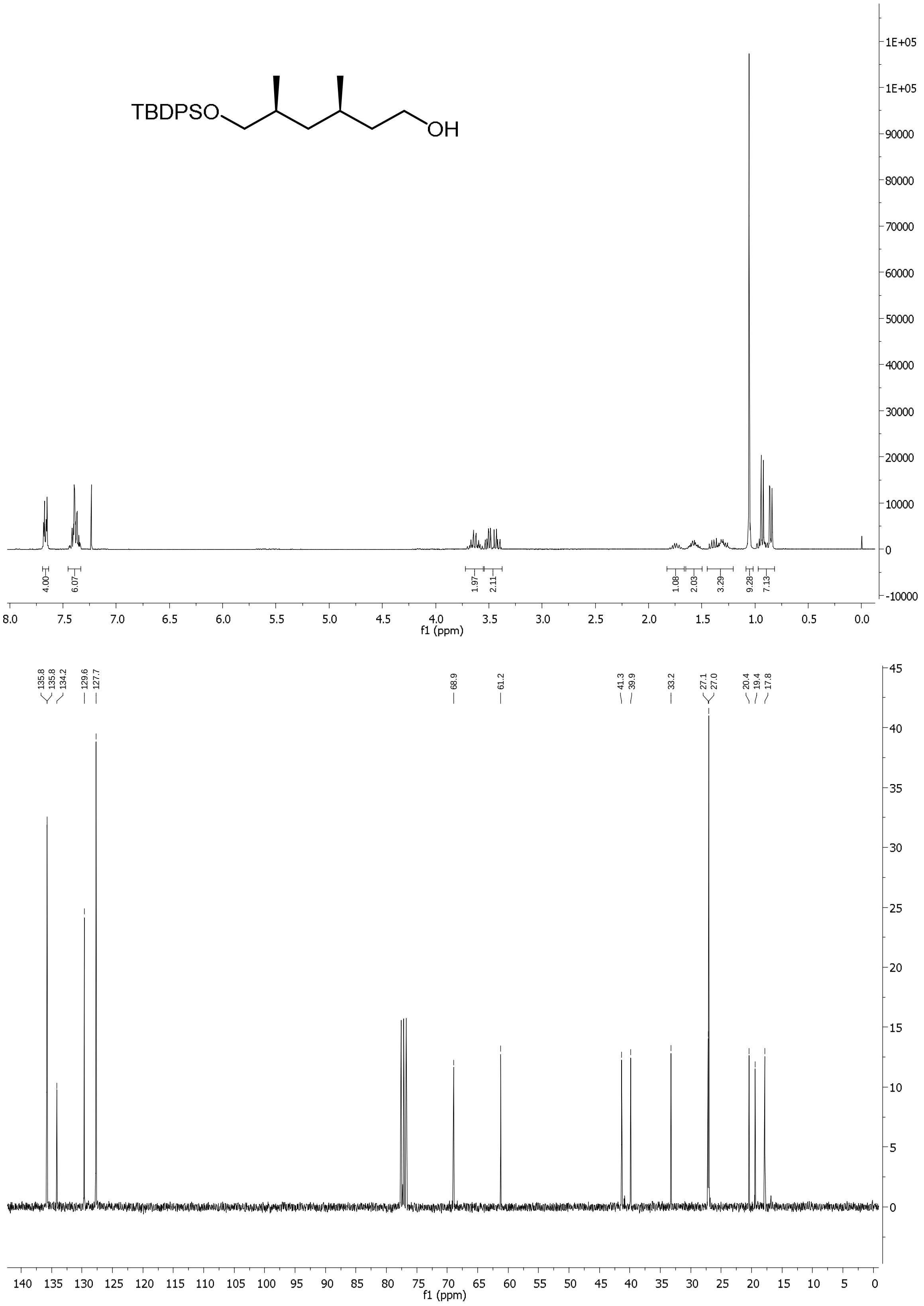

Fig. S15 ^{1}H-NMR (300 MHz, $CDCl_3$) and ^{13}C-NMR (76 MHz, $CDCl_3$) spectra of (3*R*,5*S*)-6-((*tert*-butyldiphenylsilyl)oxy)-3,5-dimethylhexan-1-ol (**5**).

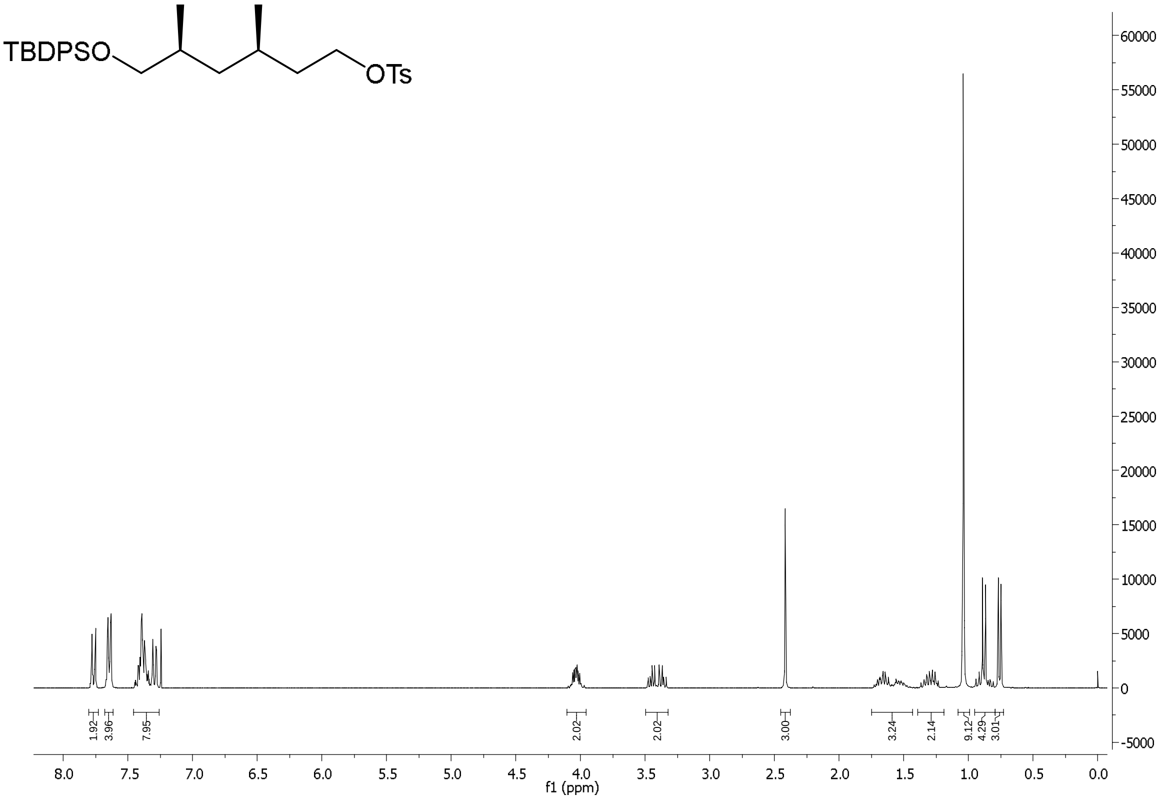

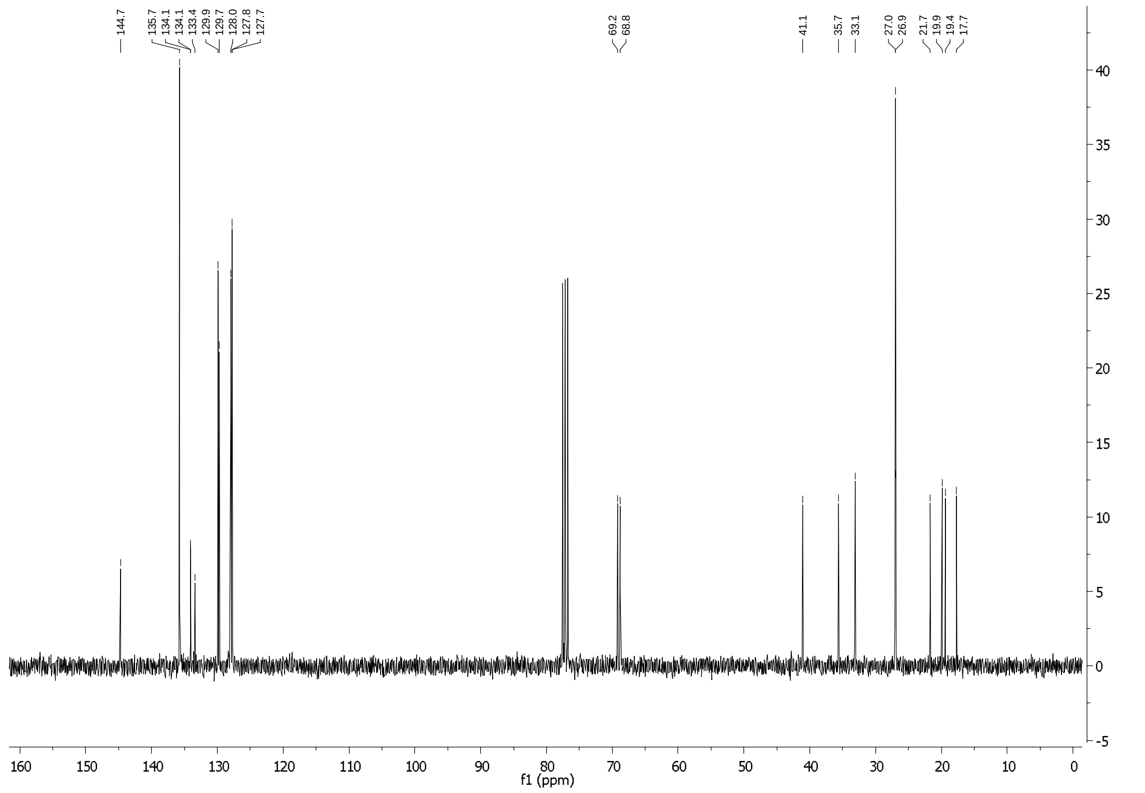

Fig. S16 ^{1}H-NMR (300 MHz, $CDCl_3$) and ^{13}C-NMR (76 MHz, $CDCl_3$) spectra of (3*R*,5*S*)-6-((*tert*-butyldiphenylsilyl)oxy)-3,5-dimethylhexyl 4-methylbenzenesulfonate (**6**).

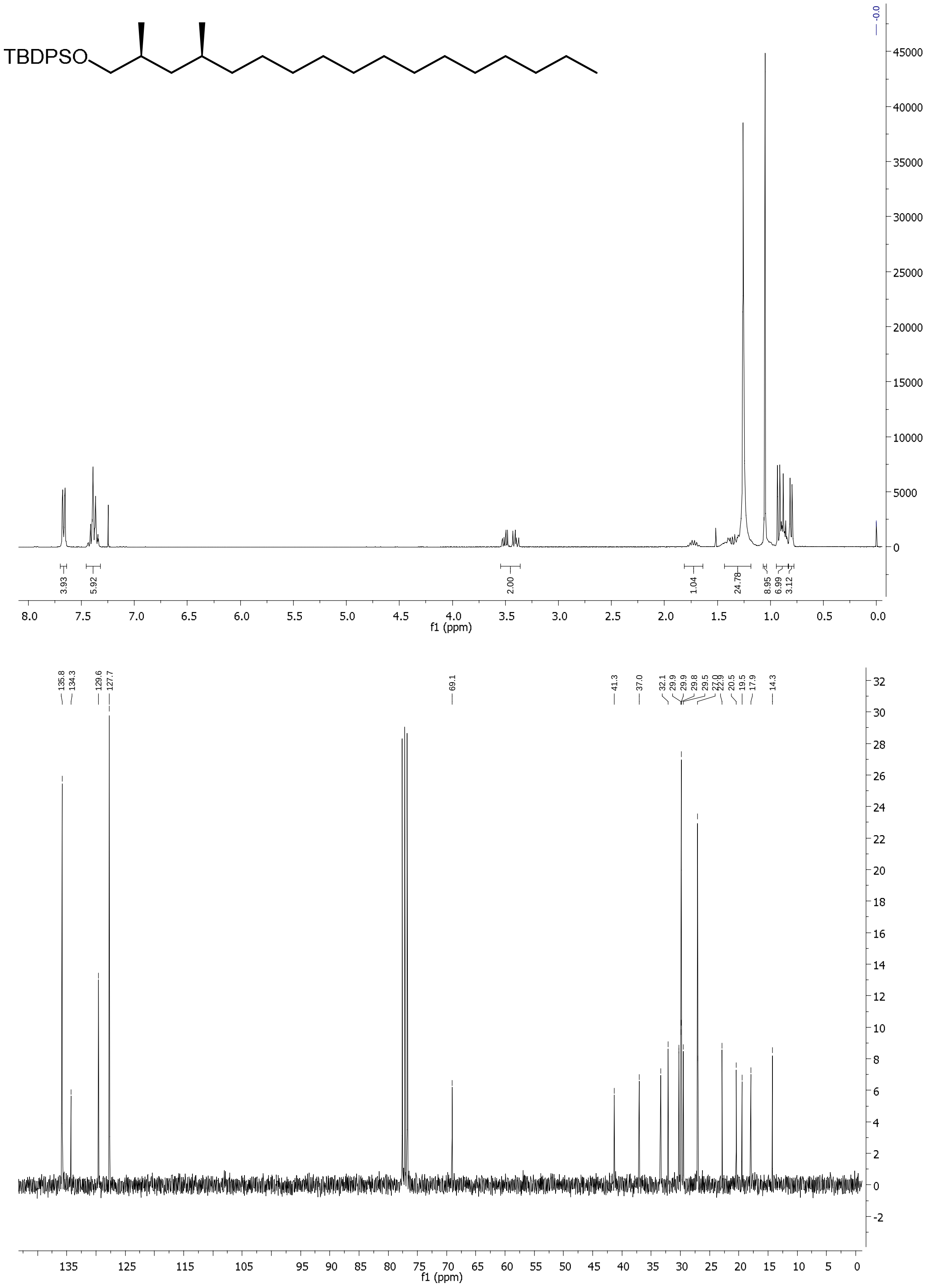

Fig. S17 ^{1}H-NMR (300 MHz, $CDCl_3$) and ^{13}C-NMR (76 MHz, $CDCl_3$) spectra of *tert*-butyl(((2*S*,4*S*)-2,4-dimethylheptadecyl)oxy)diphenylsilane (**7**).

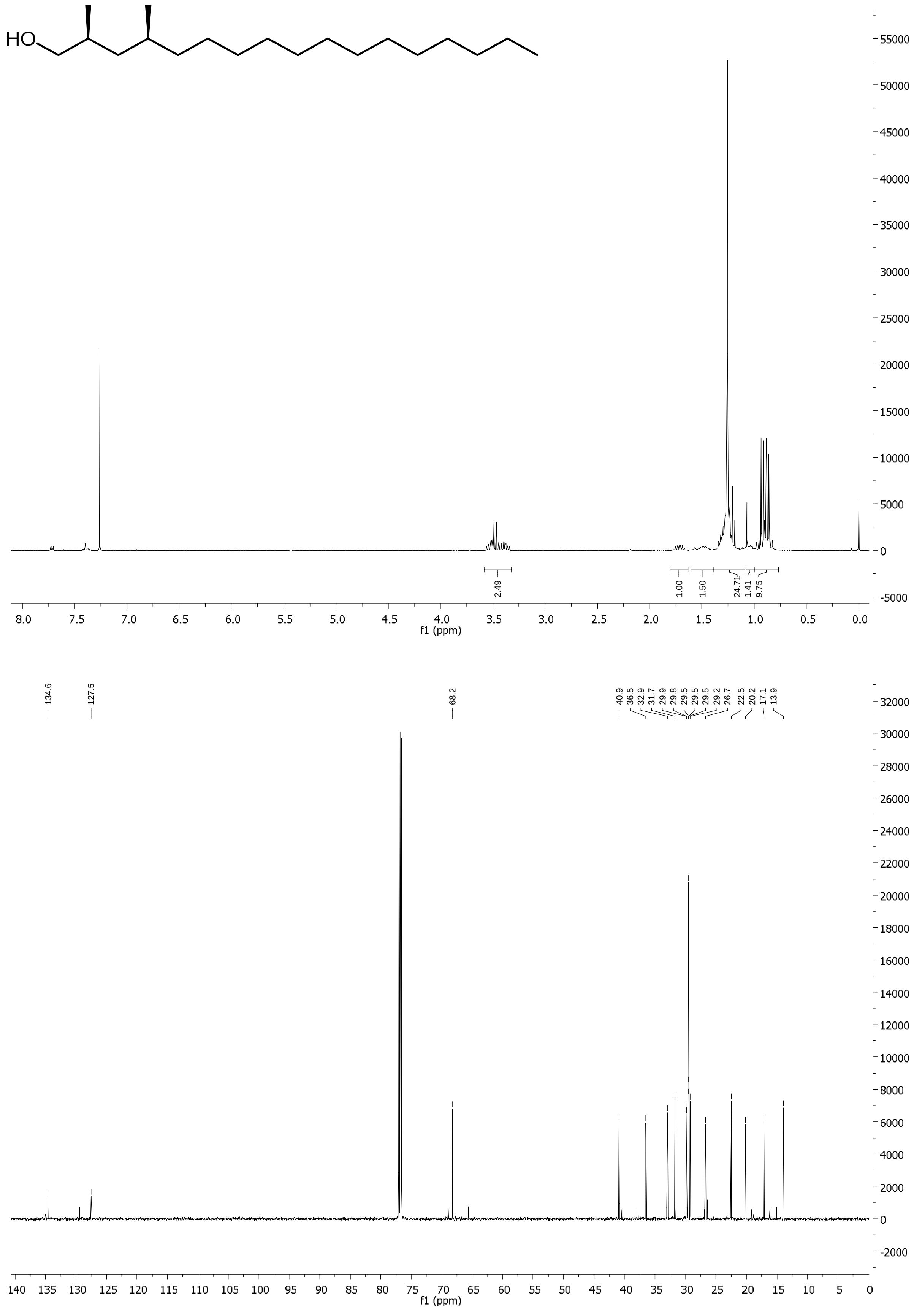

Fig. S18 ^{1}H-NMR (300 MHz, $CDCl_3$) and ^{13}C-NMR (76 MHz, $CDCl_3$) spectra of (2*S*,4*S*)-2,4-dimethylheptadecan-1-ol (**8**).

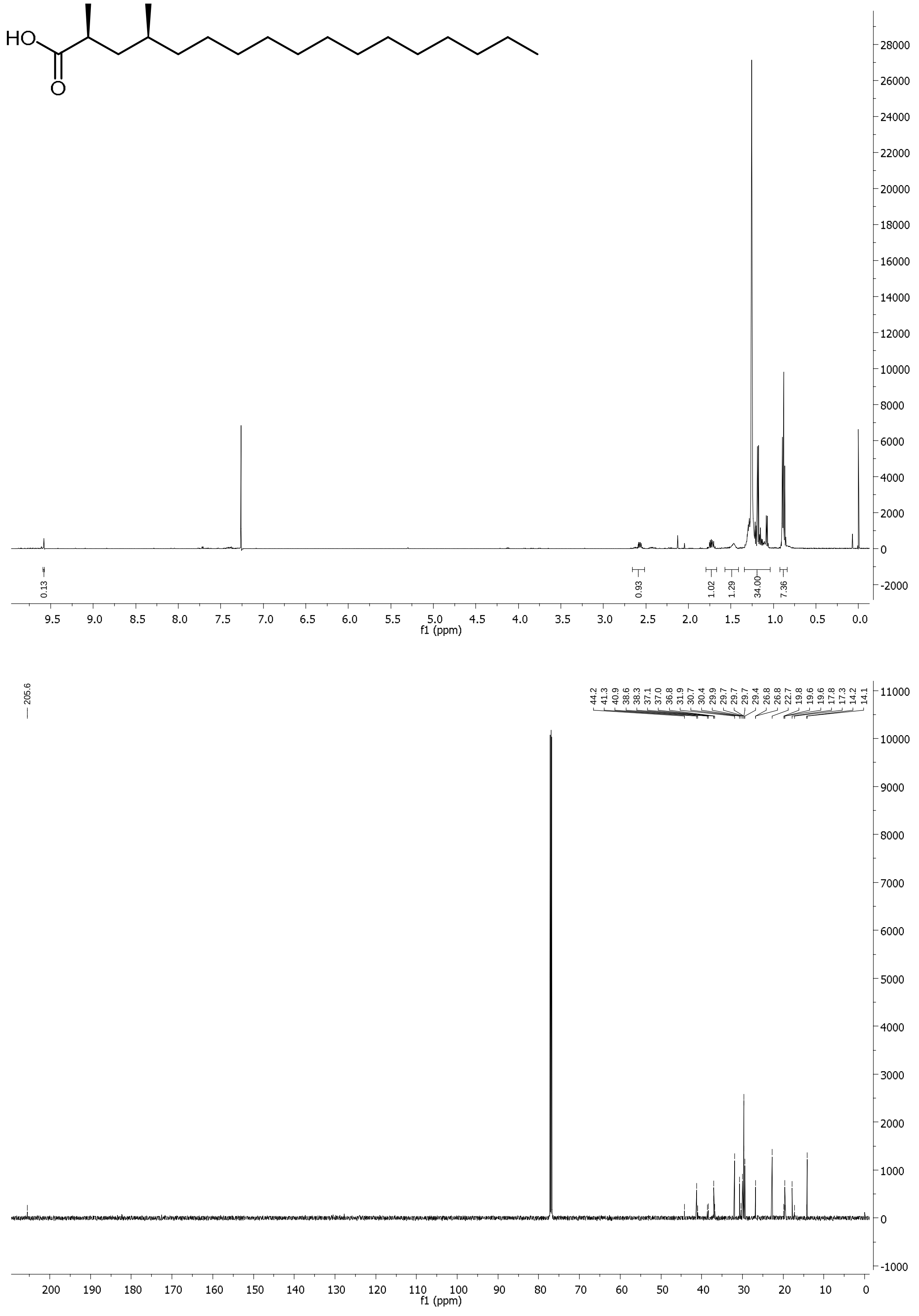

Fig. S19 ^{1}H-NMR (600 MHz, $CDCl_3$) and ^{13}C-NMR (151 MHz, $CDCl_3$) spectra of (2*S*,4*S*)-2,4-dimethylheptadecanoic acid (**9**).

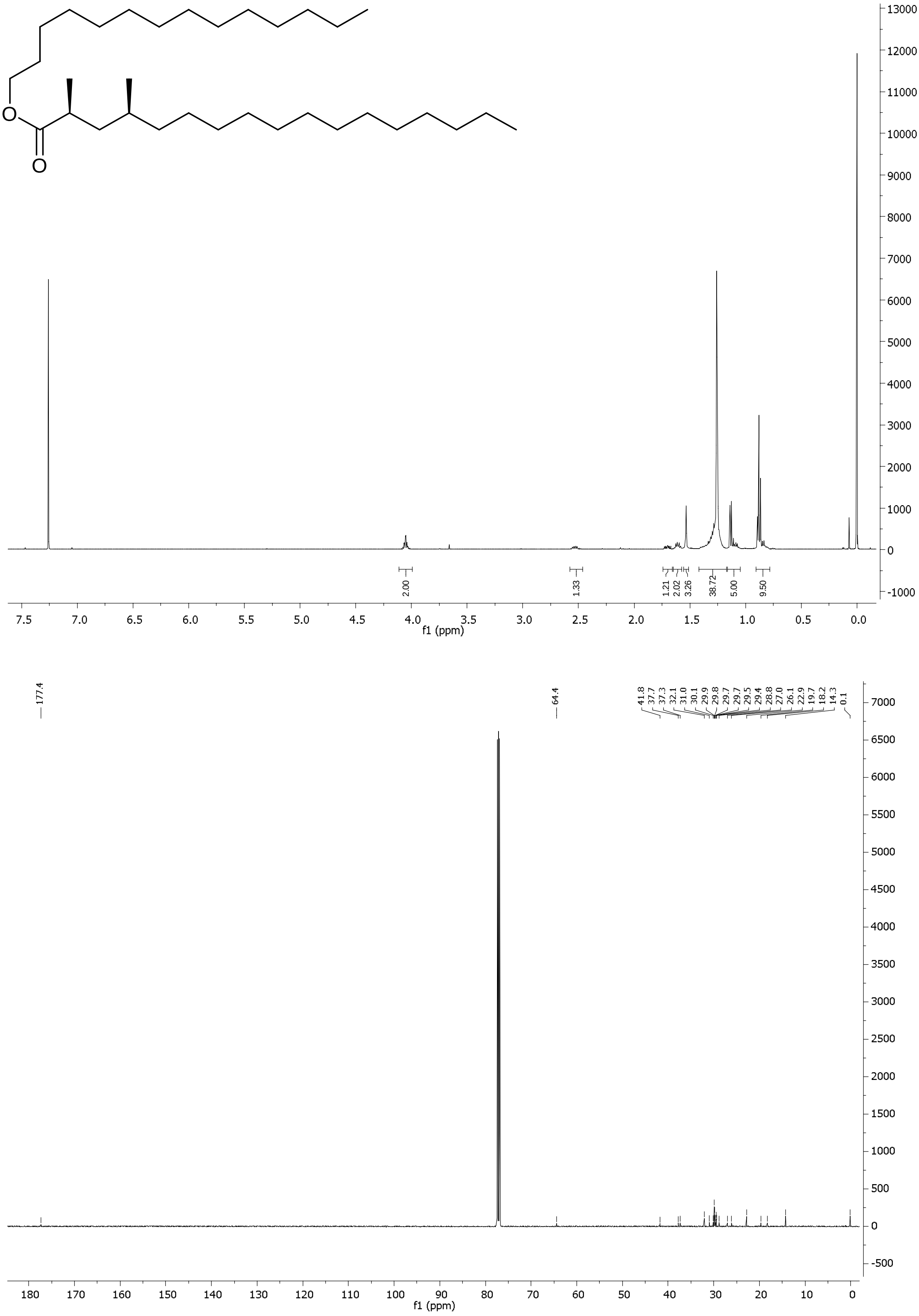

Fig. S20 ^{1}H-NMR (600 MHz, $CDCl_3$) and ^{13}C-NMR (151 MHz, $CDCl_3$) spectra of tetradecyl (2*S*,4*S*)-2,4-dimethylheptadecanoate (**11**).

Literature

Horst B ter, Feringa BL, Minnaard AJ (2007) Catalytic Asymmetric Synthesis of Phthioceranic Acid, a Heptamethyl-Branched Acid from *Mycobacterium tuberculosis*. Org Lett 9:3013–3015. https://doi.org/10.1021/ol071078o

Morgan ED (2010) Biosynthesis in insects. 2nd ed. Royal Society of Chemistry, Cambridge

Schomburg D, Michal G (2012) Biochemical pathways: An atlas of biochemistry and molecular biology. 2nd ed. John Wiley & Sons, Hoboken, N.J.

Thielman JR, Sherman DH, Williams RM (2020) Stereoselective Synthesis of Baulamycin A. J Org Chem 85:3812–3823. https://doi.org/10.1021/acs.joc.9b03443
